# Conducting molybdenum sulfide/graphene oxide/polyvinyl alcohol nanocomposite hydrogel for repairing spinal cord injury

**DOI:** 10.1186/s12951-022-01396-8

**Published:** 2022-05-06

**Authors:** Lingling Chen, Wanshun Wang, Zefeng Lin, Yao Lu, Hu Chen, Binglin Li, Zhan Li, Hong Xia, Lihua Li, Tao Zhang

**Affiliations:** 1 Guangdong Key Lab of Orthopedic Technology and Implant Materials, Key Laboratory of Trauma & Tissue Repair of Tropical Area of PLA, Orthopedic Center, General Hospital of Southern Theater Command of PLA, Guangzhou, 510010 Guangdong China; 2 The Second Clinical Medical College, Guangzhou University of Chinese Medicine, Guangzhou, 510405 Guangdong China; 3 Southern Medical University, 1023 South Shatai Road, Guangzhou, 510515 Guangdong China; 4 Department of Orthopedics, Clinical Research Centre, Zhujiang Hospital, Southern Medical University, 253 Gongye Road, Guangzhou, 510282 Guangdong China; 5 Department of Applied Physics, The Hong Kong Polytechnic University, Kowloon, 999077 Hong Kong, China

**Keywords:** Spinal cord injury, Molybdenum sulfide/oxidized graphene, Hydrogel, Conductive and mechanical adaptation, Spinal cord reparation, Anti-inflammation

## Abstract

A sort of composite hydrogel with good biocompatibility, suppleness, high conductivity, and anti-inflammatory activity based on polyvinyl alcohol (PVA) and molybdenum sulfide/graphene oxide (MoS_2_/GO) nanomaterial has been developed for spinal cord injury (SCI) restoration. The developed (MoS_2_/GO/PVA) hydrogel exhibits excellent mechanical properties, outstanding electronic conductivity, and inflammation attenuation activity. It can promote neural stem cells into neurons differentiation as well as inhibit the astrocytes development in vitro. In addition, the composite hydrogel shows a high anti-inflammatory effect. After implantation of the composite hydrogel in mice, it could activate the endogenous regeneration of the spinal cord and inhibit the activation of glial cells in the injured area, thus resulting in the recovery of locomotor function. Overall, our work provides a new sort of hydrogels for SCI reparation, which shows great promise for improving the dilemma in SCI therapy.

**Graphical Abstract:**

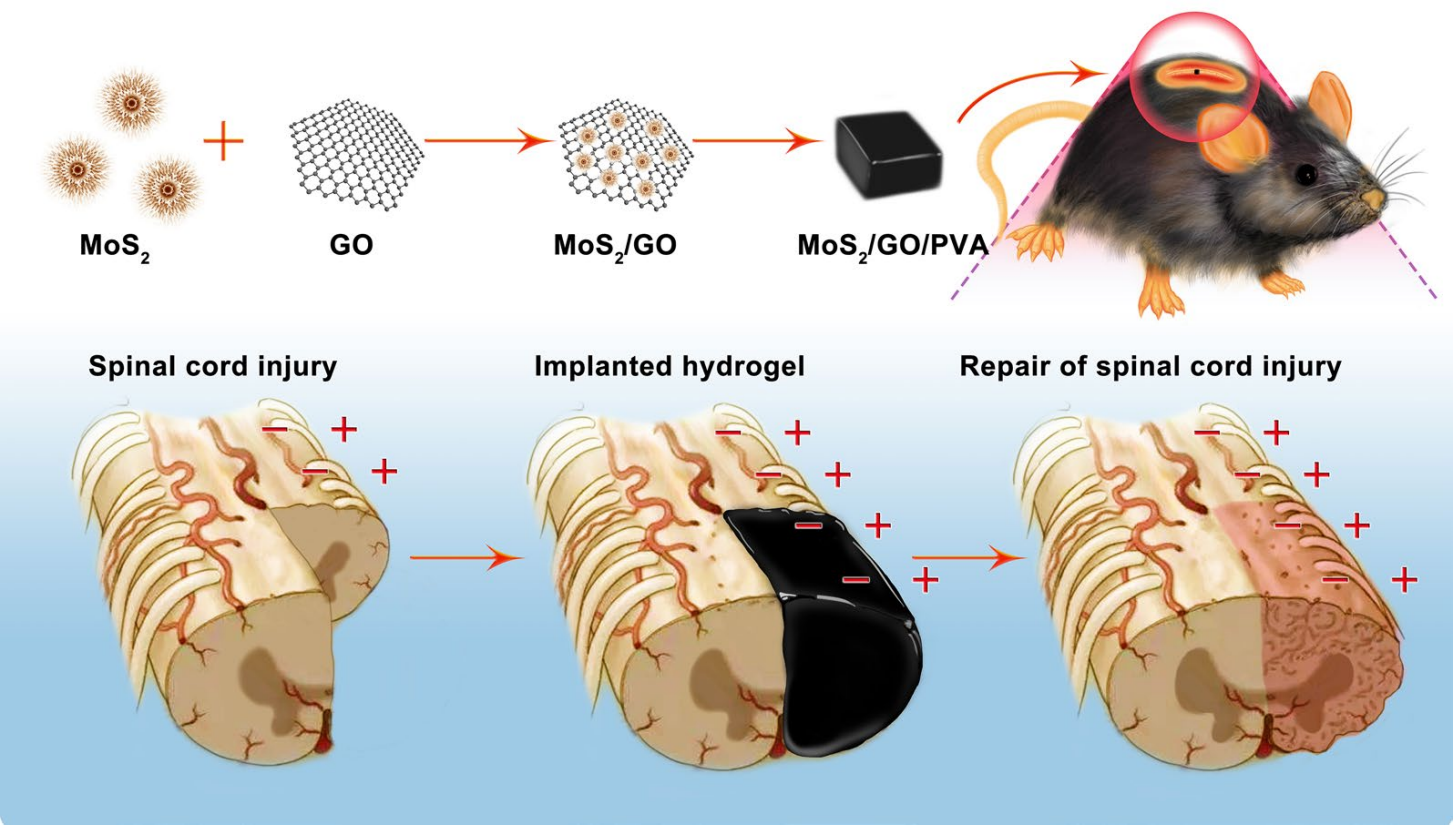

**Supplementary Information:**

The online version contains supplementary material available at 10.1186/s12951-022-01396-8.

## Introduction

Spinal cord injury (SCI), as a destructive central nervous system (CNS) trauma, is an intractable medical challenge, which could lead to motor dysfunction and paralysis for a lifetime [1]. According to the World Health Organization (WHO), about 250,000–500,000 people suffer from SCI each year worldwide [2]. And the global burden of SCI is gradually increasing because of traffic accidents [3]. Although enormous efforts have been made for SCI treatment during the past years, there is still a lack of effective treatment for SCI recovery due to the rigid microenvironment and limited regeneration ability of the central nervous system [4]. Engineering implants with high biological activity and conductivity open new avenues for improving SCI recovery.

Bioactive materials with conductive properties can mediate the signal transduction between cells and promote trauma recovery. In recent years, two-dimension (2D) nanomaterials, including graphite oxide (GO) and molybdenum sulfide (MoS_2_), have attracted much attention as new bioactive materials. GO has been used for the study of SCI because of its unique physical and chemical properties, including large loading volume, hydrophilic functional groups, and excellent biocompatibility [5]. Recent studies have also shown that GO not only facilitated the differentiation of neural stem cells (NE-4C) to neuron cells, but also promoted the directional growth of neuronal axons during the repair of SCI [6, 7]. Notably, the primary injury in SCI damages cells and initiates a complex secondary injury cascade local hemorrhage, edema, ischemia, internal environment disorders, excessive reactive oxygen species (ROS) production, and the inflammatory factors, which will further lead to nerve cell damage [8] and destroy the connection of neurons [9].

MoS_2,_ with high electrical conductivity and catalytic activity, is preferred as doped materials [10]. Previous studies suggested that MoS_2_ promoted anti-inflammatory, macrophage-modulating SCI immunotherapy and provided neuro-reconnection and protection, resulting in locomotor recovery correspondingly [11]. Hence, MoS_2_/GO displays great potential for SCI repair. More importantly, materials for renovation should have high elasticity to eliminate the mechanical stress on the spinal cord and low friction coefficient [12, 13].

**Scheme 1.**
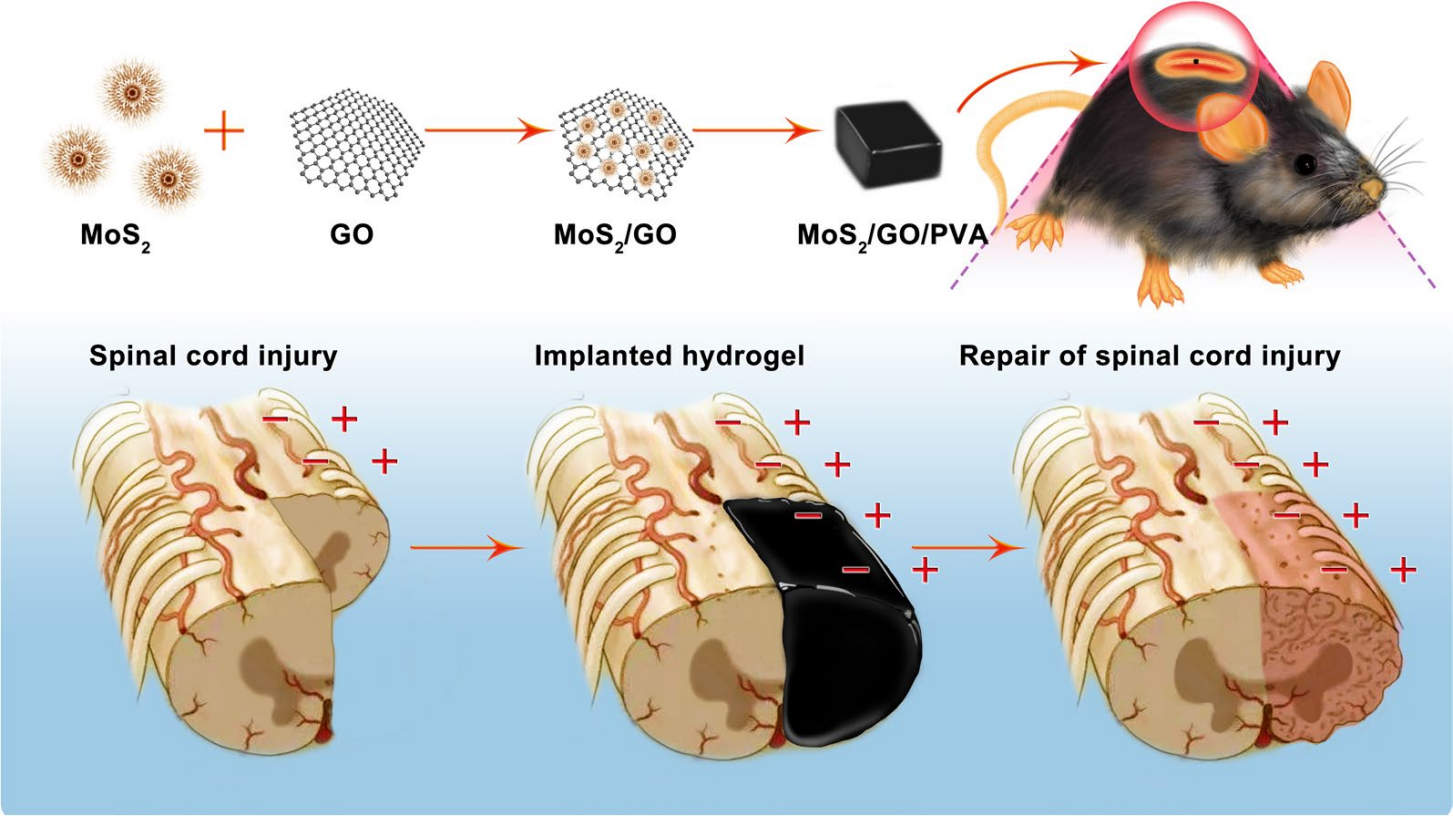
The schematic diagram shows the preparation steps of MoS_2_/GO/PVA nanocomposite hydrogel and the treatment process of mouse SCI

To address the dilemma in SCI therapy, we proposed polyvinyl alcohol PVA hydrogels with MoS_2_/GO nanosheets (NSs). PVA is a promising candidate because it can prevent the migration of inflammatory cells and reduce secondary injury after SCI. The synthesized composite hydrogel exhibits good suppleness, proper Young’s modulus, and high electrical conductivity. They promoted the directional differentiation of neural stem cells into neuron cells and scavenged reactive oxygen species (ROS) in the injured part. In addition, the composite hydrogel can inhibit the differentiation of M1 and activate M2 macrophage, thus resulting in the improvement of inflammatory cytokines (Scheme 1). More importantly, MoS_2_/GO/PVA composite hydrogels could remarkably promote spinal cord tissue repair and locomotor recovery in vivo. Therefore, MoS_2_/GO/PVA composite hydrogel works as an excellent biomaterial for the treatment of SCI and has a promising clinical application prospect.

## Materials and methods

### Composite hydrogel synthesis

Firstly, GO NSs were prepared from graphite powder. A large beaker was placed on ice, 110 mL of H_2_SO_4_ (98%) was added, then 5 g graphite was added at 4 °C, and 2.5 g NaNO_3_ and 15 g KMnO_4_ were slowly added in order, and the solution was purple green after 90 min of stirring on a magnetic stirrer. The ice was removed, and the mixture was reacted for another 30 min. The solution was slowly added to 220 mL ddH_2_O and heated to 70 °C, then 20 mL H_2_O_2_ (5%) was slowly added to the solution. At this time, the solution turned golden. After the reaction, the sample was centrifugated and washed for several times to remove the overabundant acid. The sample was dried at 80 °C, and the GO NSs were obtained after grinding.

Synthesis of MoS_2_ and MoS_2_/GO NSs, 0.6 g sodium molybdate was dissolved in 70 mL ddH_2_O, 0.78 g thiourea and 0.1 g polyvinyl pyrrolidone (PVP) were added to the solution, after ultrasonicated for 20 min, the solution was transferred into a 100 mL Teflon-lined autoclave, sealed, and heated up to 240 °C for 24 h in an electric furnace. After the reaction naturally fell to room temperature, the precipitates were got from centrifugation at 8000 rpm for 10 min and then washed with ethanol and distilled water to remove the impurities. Similarly, the MoS_2_/GO NSs were synthesized with the abovementioned methods after replacing the PVP with GO. Additionally, all the MoS_2_ NSs were closely attached on the GO NSs during the hydrothermal process.

Synthesis of GO/PVA, MoS_2_/PVA, and MoS_2_/GO/PVA composite hydrogel. The PVA concentration was set to 2.5%, 5%, 7.5%, 10%, 12.5%, and 15% (w/v), respectively. After repeatedly freezing and thawing overnight at −20 °C, composite hydrogels with different properties were obtained. The preparation of GO/PVA, MoS_2_/PVA, and MoS_2_/GO/PVA composite hydrogel were as below, 100 mL MoS_2_, GO and MoS_2_/GO solutions (125 μg/mL) were mixed with 100 mL PVA (2 g/mL), respectively, and the different solutions were repeatedly freezing and thawing overnight at −20 °C to get 62.5 μg/mL GO/PVA, MoS_2_/PVA, and MoS_2_/GO/PVA composite hydrogel.

### Characterization

The morphology of the nanomaterials was observed by high-resolution transmission electron microscopy (HRTEM, JEOL 2100, Japan) after MoS_2_, GO, and MoS_2_/GO NSs suspensions were placed on a carbon-coated copper net. The surface morphology of the hydrogel was observed using a scanning electron microscope (Nova Nano SEM 430, FEI). Raman spectra of MoS_2_, GO, and MoS_2_/GO NSs were recorded using a confocal Raman microscope (Labram HR, Horiba Jobin Yvon, France). The X-ray diffraction (XRD) patterns of MoS_2_/GO and MoS_2_ were obtained using the Siemens Kristalloflex 810 D-500 X-ray diffractometer (Karlsruhe, Germany) in the 2θ range of 5° to 90° at 40 kV and 30 mA, λ = 1.5406 A radiation. Fourier transform infrared (FTIR) spectra were measured on a PerkinElmer 580B IR spectrophotometer using the KBr pellet technique. The high-angle annular dark-field scanning TEM (HAADF-STEM) image and element mapping were obtained from the electron microscope (Hitachi 2100, Japan) with an accelerating voltage of 210 kV. The x-ray photoelectron spectroscopy (XPS) spectra was applied to determine the element type and chemical valence state of the MoS_2_/GO NSs using the electron spectrometer (ESCALAB 250Xi, Thermo Scientific, USA). The Zeta potential of MoS_2_, GO, and MoS_2_/GO NSs were measured by Zetasizer Nano Z (Malvern, UK).

### Peroxidase-like activity

Peroxidase (POD)-like activity of GO, MoS_2_, and MoS_2_/GO NSs was evaluated using 3,3',5,5'-tetramethylbenzidine (TMB, Macklin, China) according to our previous reports [14]. For the oxidation of TMB by H_2_O_2_, three groups with various reagents combinations were set as followed: (1) TMB + H_2_O_2_, (2) TMB + GO or MoS_2_ or MoS_2_/GO NSs, (3) TMB + H_2_O_2_ + GO or MoS_2_ or MoS_2_/GO NSs. 100 μL of GO or MoS_2_ or MoS_2_/GO NSs (100 μg/mL) were suspended in acetic acid-sodium acetate buffer (0.2 M:0.2 M, pH = 4.6), respectively. Then, 100 μL of TMB solution in dimethyl sulfoxide (2 mM) as substrate, or 100 μL of H_2_O_2_ (10 mM) were added. During half-hour incubation at room temperature, changes in the UV–Vis absorption spectra were monitored using the UV–Vis spectrometer every 5 mins.

### Conductivity

The conductivity of hydrogels was detected by a digital multimeter (fluke 8845A, American), the cross-section and length of hydrogels were measured by digital vernier caliper (Neiko 01407A, American) (Additional file 1: Fig. S6), and the conductivity of hydrogel was calculated comprehensively.

### Swelling ratio

To evaluate the swelling ability of nanocomposite hydrogels, the weight of the dried hydrogels and wet hydrogels after being immersed in 37 °C phosphate buffer saline (PBS) for 24 h were recorded, respectively. The swelling rate was determined by using the following equation:

1 $$ {\text{Swelling rate }}\left( \% \right) \, = \frac{{\text{Wet weight - Dry weight}}}{{\text{Dry weight}}} \times 100 $$

### Biodegradation

Simulated body fluid (SBF) was used to detect the biodegradation of nanohydrogels. After the initial weight were weighed, the nanohydrogels with a volume of about 0.5 cm^3^ were immersed in 10 mL SBF and placed in 15 ml centrifuge tubes, and stirred at a constant speed of 100 rpm in 37 °C. 10% PVA hydrogel was used as Control group. SBF was replaced once every two days, and the nanohydrogels weight was measured on day 1, 3, 5, 7, 14, 21, 28, 35 and 42 after the surface moisture was dried with absorbent paper. The degradation (%) was determined by using the following equation:

2 $$ {\text{Degradation}}\left( \% \right) \, = \frac{\text{Final weight}}{\text{Initial weight}} \times 100 $$

### Cell culture

Mice neural stem cells (NE-4C, ATCC, CRL-2925) and human neuroblastoma cells (SH-SY5Y, ATCC, CRL-2266) were cultured in low glucose DMEM (Gibco; Thermo Scientific, USA) medium containing 10% FBS (VivaCell, Shanghai, China) and penicillin (100 U/mL) and streptomycin (100 μg/mL) (Invitrogen). Cells were incubated in an incubator at 37 °C and 5% CO_2_. The medium was replaced every other day.

### Cytotoxicity of nanocomposite hydrogels

The cytotoxicity of nanocomposite hydrogels against NE-4C was assessed using Cell Counting Kit 8 (CCK-8) (Dojindo Laboratories, Kumamoto, Japan). NE-4C cells were seeded in 96-well plates with a density of 4 × 10^4^ cells per well. Different concentrations of MoS_2_, GO, and MoS_2_/GO NSs (500, 250, 125, 62.5, 31.25, 15.6, 7.8, 0 μg/mL) were added to cells after 24 h. After cocultured for another 24 h, the CCK-8 assay was taken to evaluate their toxicity. Additionally, the proliferation rate of PVA, MoS_2_/PVA, GO/PVA, and MoS_2_/GO/PVA hydrogels was measured using CCK-8 assay after cocultured with NE-4C cells for 24 h.

### Live/dead staining

0.1 g PVA, MoS_2_/PVA, GO/PVA, and MoS_2_/GO/PVA hydrogels were firstly added to the 24-well plate, then NE-4C cells were seeded in the wells with a density of 1 × 10^5^ cells. After 24 h culture, a live/dead staining kit (BestBio, Shanghai, China) was applied to different treatment groups, and the images were obtained using a Leica DMI3000B inverted microscope (Leica, Germany). The quantitative analysis of living and dead NE-4C cells was conducted by ImageJ2x (Rawak Software, Germany).

### Hemolysis tests

5 mL Blood was obtained from the mice. Blood was placed in a triangular bottle with glass beads and slightly stirred to remove fibrin. Then the blood was centrifuged with normal saline till the supernatant was colorless and transparent, and then the washed red blood cells were mixed with normal saline into 2% of cell suspension for later use. GO/PVA, MoS_2_/PVA, and MoS_2_/GO/PVA were prepared with normal saline at concentrations of 500, 250, 125, and 62.5 μg/mL, and 10 mg of composite hydrogels were immersed in 0.8 mL normal saline for incubation at 37 °C for 1 h. 0.8 mL ddH_2_O and PBS were set as positive control and negative control, respectively. Afterwards, 0.2 mL of diluted erythrocyte suspension was added to each sample and incubated at 37 °C for 1 h. After that, all samples were centrifuged at 3000 rpm for 5 min and the supernatant was collected. The O.D. of the supernatant was measured at 545 nm using a microplate analyzer. The hemolytic percentage was calculated using the following formula:

3 $$ {\text{Hemolysis rate }}\left( \% \right) \, = \frac{{{\text{ O}}.{\text{D}.}_{{{\text{Sample}}}} {\text{ - O}}.{\text{D}.}_{{\text{Negative control}}} }}{{{\text{O.D}}{.}_{{\text{Positive control}}} {\text{ - O}}{\text{.D}}{.}_{{\text{Negative control}}} }} \times 100 $$

### Neural cell differentiation

In order to make the cells better enter the state of preparation for the following induction of differentiation, we changed the medium of NE-4C and SH-SY5Y cells into serum-free DMEM/F12 (Gibco, Thermo Scientific, USA) containing 1% penicillin–streptomycin, 1% B-27 supplement, and 5 μM retinoic acid (RA). The NE-4C and SH-SY5Y cells were respectively cocultured with different hydrogels for 3 and 7 days, and then the phenotype of neural cell differentiation was detected by immunofluorescence and PCR.

### Immunostaining fluorescence

0.1 g PVA, MoS_2_/PVA, GO/PVA, and MoS_2_/GO/PVA hydrogels were firstly added to the 24-well plate, then NE-4C cells were seeded in the wells with a density of 1 × 10^5^ cells. After being cocultured with different hydrogels for 3 and 7 days, the cells were fixed with 4% formaldehyde on ice for 15 min. After being washed with PBS, the cells were incubated in 0.5% TritonX-100 and diluted by PBS at room temperature for 20 min. After being washed with PBS, the cells were sealed at room temperature in 5% of BSA for 1 h. Then, primary antibodies anti-Tuj1 (GTX108731, 1:500; Genetex) and GFAP (GTX108711, 1:500; Genetex) diluted in 1% of BSA solution was added to the cells and incubated overnight at 4 °C. The cells were rewashed with PBS and incubated with DyLight 488 goat anti-mouse IgG (diluted 1000 times in 5% BSA, Biodragon) and Alexa Fluor 633 binding goat anti-rabbit IgG (diluted 1000 times in 5% BSA, Biodragon). After being incubated for 30 min at 37 °C in a dark environment, the secondary antibodies were washed. The nuclei were stained with DAPI dye, and the images were captured using a ZEISS LSM 900 confocal microscope (ZEISS, Germany).

### Gene expression analysis

The RNA in PVA, MoS_2_/PVA, GO/PVA, and MoS_2_/GO/PVA hydrogels treated groups was extracted by RNA extraction kit (R4114-02, Magen, China), and RNA was reversely transcribed into cDNA by reverse transcription kit (11939823001, Roche, Switzerland). RT-PCR was performed using Maxima TM SYBR Green/Rox qPCR Master Mix (4710436001, Roche, Switzerland). Each individual PCR assay was repeated at least three times, and the expression of GAPDH was used to calculate the expression level of each detected gene. 2^–ΔΔ^ quantitative Ct method was used to calculate relative gene expression. The primers used in this study are listed in Table 1.

**Table 1:** Primers sequence used for real-time PCR

Target	Forward	Reverse
GAPDH	AGGTCGGTGTGAACGGATTTG	GGGGTCGTTGATGGCAACA
GFAP	CGGAGACGCATCACCTCTG	TGGAGGAGTCATTCGAGACAA
HB9	GAACACCAGTTCAAGCTCAACA	GCTGCGTTTCCATTTCATTCG
Islet-1	TTTCCCTGTGTGTTGGTTGC	TGATTACACTCCGCACATTTCA
ChAT	GGCCATTGTGAAGCGGTTTG	GCCAGGCGGTTGTTTAGATACA
Tuj1	TAGACCCCAGCGGCAACTAT	GTTCCAGGTTCCAAGTCCACC
IL-10	TTGTCGCGTTTGCTCCCATT	GAAGGGCTTGGCAGTTCTG
IL-6	TGGGGCTCTTCAAAAGCTCC	AGGAACTATCACCGGATCTTCAA
TNF-α	CAGGCGGTGCCTATGTCTC	CGATCACCCCGAAGTTCAGTAG

### ROS detection in NE-4C cells

ROS in NE-4Cs after 24 h seeded on GO/PVA, MoS_2_/PVA and MoS_2_/GO/PVA hydrogels was detected by reactive oxygen species detection kit (ab113851, Abcam, China), in which DCFH-DA was diluted to the final concentration of 10 μmol/L in serum-free medium at 1:1000. The cell culture medium was removed, and 200 μL DCFH-DA (1:1000) was added. The fluorescence images were captured using a fluorescence microscope (DMI3000B, Leica, Germany).

### Flow cytometry

0.1 g PVA, MoS_2_/PVA, GO/PVA, and MoS_2_/GO/PVA hydrogels were firstly added to the 24-well plate, then NE-4C cells were seeded in the wells with a density of 1 × 10^5^ cells. Then 100 ng/mL lipopolysaccharide (LPS) (L4391-1 mg, Sigma-Aldridge) were added to the culture for 24 h, and cells were then collected, washed twice with cold PBS, stained with FITC anti-mouse CD206 (141703 Biolegend, USA) and PE anti-mouse CD11c (101307, Biolegend, USA) in the binding buffer for 30 min, and then detected by flow cytometry (FACS Calibur flow cytometry; BD Biosciences). FlowJo-V10 software was used to analyze the proportion of M1and M2 macrophages in each group.

### SCI model in mice

All aspects of animal experiments were approved by the Ethical Committee of Laboratory Animals of General Hospital of Southern Theater Command of PLA. Male C57BL/6 N mice were aged 6–8 weeks old and purchased from Guangdong Medical Laboratory Animal Center. The animals were randomly divided into five groups: Sham, SCI, GO/PVA, MoS_2_/PVA, and MoS_2_/GO/PVA (n = 15). C57 mice were anesthetized by intraperitoneal injection with 4 mL/kg 10% chloral hydrate. After skin preparation, iodophor was used to disinfect the skin of the operation area. Skin soft tissues were cut at the T9-10 level to expose the spine, and the lamina was cut to expose the spinal cord. 2 mm of the spinal cord was removed on the right side. The hydrogels of the same size were implanted. The surgical incision was sutured layer by layer, only the spinal cord was exposed in the Sham operation group, and no hydrogel was implanted after spinal cord resection in the SCI group.

### Histological staining

Six weeks after the operation, the C57 mice were sacrificed by neck-breaking, the surgical area and the surrounding spinal cord were removed, the tissue samples were quickly fixed with 4% paraformaldehyde for 24 h, and then washed with PBS for three times and 50% alcohol for twice, followed by ethanol gradient dehydration and paraffin-embedded tissues. The xylene transparent tissues were repaired, and the embedded tissue block was cut into paraffin tape of 4 μm with a slicer. After being stained with hematoxylin and eosin (H&E), the images were observed under Olympus BX51 light microscope.

### Immunohistochemistry staining

Immunohistochemistry was used to evaluate the effects of nanocomposite hydrogels on the differentiation of neonatal spinal cord tissue in vivo. Tissue sections from the same wax block used for H&E staining were taken for immunohistochemistry staining. After routine dewaxing and rehydration, the antigen was repaired with 3% H_2_O_2_. They were sealed with goat serum at 37 °C for 30 min. The samples were then incubated overnight with primary antibodies: rabbit anti-HB9 antibody (ORB157435, 1:1000, Biorbyt), rabbit anti-Islet-1 antibody (GTX 102807, 1:1000, Genetex), rabbit anti-ChAT antibody (PA5, 1:10000, Thermo Fisher), rabbit anti-GFAP antibody (GTX108711, 1:1000, Genetex), and rabbit anti-Tuj1 (GTX130245, 1:1000, Genetex) at 4 °C. After being washed with PBS, the samples were incubated with goat anti-rabbit IgG secondary antibody (ab6747, Abcam, China) at 37 °C for 10 min. The being colorized by the chromogenic agent, the sections were re-dyed with hematoxylin and eosin. They were dehydrated, sealed, observed, and photographed under a light microscope (Olympus BX51, Japan).

### Functional assessment

Basso, Beattie and Bresnahan (BBB) score was applied to evaluate the recovery of hind limb motor function in mice. Firstly, mice were placed in an open and flat place, and their movements were observed and recorded. The full score of the three items was 21 points, which were evaluated by the movements of the hind limbs, the gait and coordination function of the hind limbs, and the fine movements of the claws in the movement. The animals were evaluated weekly. The graders were conducted by three people blindly. Secondly, after the front and rear feet of mice were marked with black and red dyes, they were placed in a track covered with white paper in advance to make the animals run from one end to another. The inter limb coordination of the center of the front and rear feet of mice on the same side and the angle of rotation of the third foot of the hind limb were observed for analysis.

### Western blot

The collected spinal cord tissue samples were quickly frozen in liquid nitrogen, where the tissue was ground until there were no obvious tissue particles. Electrophoresis gel was prepared using a gel preparation kit (Beyotime, Shanghai, China) to isolate total protein of spinal cord tissue. Rapid membrane transfer apparatus (Bio-Rad, USA) was used to transfer the isolated protein onto a polyvinylidene fluoride (PVDF) membrane. The PVDF membrane was sealed with 5% skim milk powder and prepared by PBS for 1 h. Then, the membrane was incubated with primary antibodies: rabbit anti-HB9 antibody (ORB157435, 1:1000, Biorbyt), rabbit anti-Islet-1 antibody (GTX 102807, 1:1000, Genetex), rabbit anti-ChAT antibody (PA5, 1:10000, Thermo Fisher), rabbit anti-GFAP antibody (GTX108711, 1:1000, Genetex), and rabbit anti-Tuj1 (GTX130245, 1:1000, Genetex) at 4 °C overnight. The horseradish peroxidase labeled sheep anti-rabbit secondary antibody (ab205718, Abcam, China) was incubated at room temperature for 1 h, and the ECL luminescence kit (WBKLS0100, Millipore, USA) was used for luminescence determination. The gray value of each strip was calculated and analyzed by using Image J software.

### Statistical analysis

All statistical analyses in this study were performed using IBM SPSS (version 23.0). All data are represented as mean ± SD, and the number of duplicate legends is represented as “n” in the legend. One-way analysis of variance (ANOVA) was used for comparison between the groups at a single time point. Comparison between the two groups at multiple time points was performed using two-way ANOVA, followed by multiple post-hoc comparisons (Dunnett-t test). * p < 0.05, ** p < 0.01.

## Results and discussion

### The synthesis and characterization of MoS_2_/GO/PVA

**Fig. 1:**
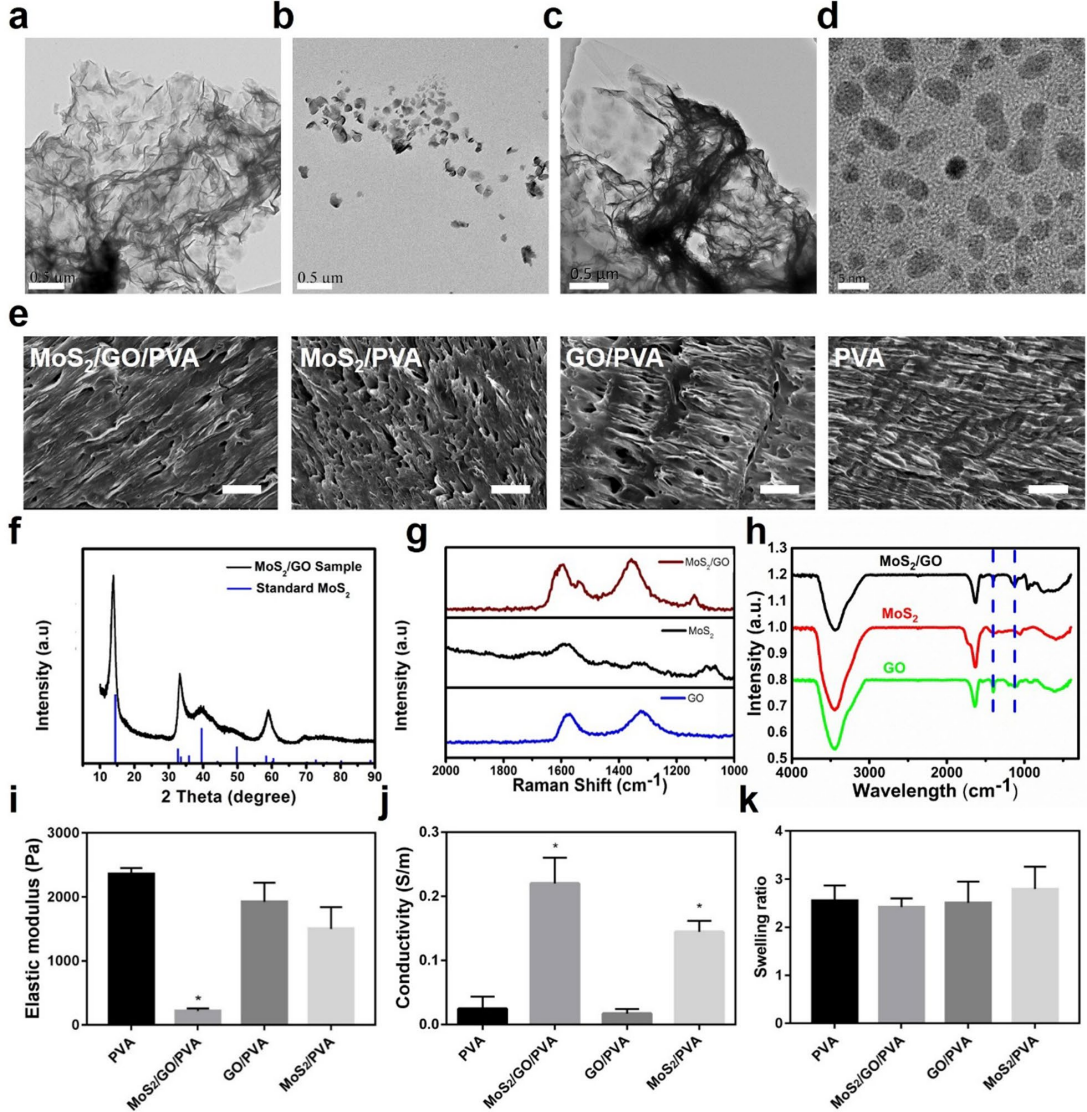
Characterization of MoS_2_, GO, and MoS_2_/GO NSs. TEM images of (**a**) GO, **b** MoS_2_, and **c**, **d** MoS_2_/GO NSs. Scale bar = 0.5 μm or 5 nm (**e**) SEM images of MoS_2_/GO/PVA, MoS_2_/PVA, GO/PVA and PVA hydrogels, ×1000 (from left to right). Scale bar = 20 μm. **f** XRD pattern of standard MoS_2_ and synthetic MoS_2_/GO sample. **g** Raman spectra of GO and MoS_2_/GO NSs. (h) FTIR spectra of GO and MoS_2_/GO NSs. **i** Elastic modulus, **j** conductivity, and **k** swelling ratio of PVA, GO/PVA, MoS_2_/PVA, and MoS_2_/GO/PVA hydrogels

Briefly, MoS_2_/GO NSs was synthesized by a hydrothermal method, and the MoS_2_/GO PVA composite hydrogel was obtained from repeated freezing and thawing. HRTEM image showed that the translucent GO (Fig. 1a), MoS_2_ (Fig. 1b), and MoS_2_/GO NSs (Fig. 1c, d). The image of MoS_2_/GO NSs at high magnification displayed that MoS_2_ NSs were scattered to GO NSs. The surface morphology and microstructure of the prepared hydrogels were analyzed by SEM (Fig. 1e). MoS_2_/GO/PVA, MoS_2_/PVA, GO/PVA, and PVA hydrogels were well arranged with gap structure. The SEM results showed that the surface of hydrogels in each group was evenly and neatly spaced, and no obvious granular substance was found, indicating that nanoparticles were well dispersed in PVA hydrogels. GO NSs, here, have good dispersibility in PVA hydrogel [15, 16] and will further better optimize various properties of hydrogels [17]. The XRD pattern (Fig. 1f) indicated that the peaks of MoS_2_/GO were well indexed to the standard MoS_2_ crystal phases (JCPDS 74-0932), suggesting that MoS_2_ NSs successfully synthesized on GO NSs. Raman spectra (Fig. 1g) of GO showed the characteristic G band at 1580 cm^−1^ and D band at 1320 cm^−1^. Correspondingly, the G band of MoS_2_/GO NSs was peaked at 1600 cm^−1^, and the D band shifted slightly to 1360 cm^−1^, which illustrated the existence of GO in MoS_2_/GO NSs. The FTIR spectra of the MoS_2_/GO, MoS_2_, and GO NSs were shown in Fig. 1h. It can be seen that the FTIR spectra of MoS_2_ are very similar to MoS_2_/GO because of their similar organic compositions (C, H, O in all samples). Briefly, the broad vibration peak at 3200–3600 cm^−1^ is attributed to the hydroxyl/H_2_O in the NSs. Furthermore, the –OH bonding could be found at 1600–1700 cm^−1^ for all samples. The stretching vibration of CH_2_ and C–O bonds could also be found in 1000–1200 cm^−1^ and 1400 cm^−1^. HAADF-STEM was employed to conduct the elemental distribution of Mo, S, C, and O elements in the nanocomposite (Additional file 1: Fig. S1) and thus proven the abovementioned chemical elements in the MoS_2_/GO NSs. In addition, X-ray photoelectron spectroscopy (XPS) was conducted to study the elemental compositions, properties, and valence states of the MoS_2_/GO. As could be seen from Additional file 1: Fig. S2, the characteristic peaks at 231.62 eV, 161.87 eV, 285.03 eV, 531.22 eV in XPS spectra were well matching to Mo 3d, S 2p, C 1 s, and O 1 s, respectively, which further confirmed that MoS_2_ NSs was anchored on GO NSs in MoS_2_/GO NSs. After a series of characterization analysis and optimization, MoS_2_ and GO with the molar ratio of 1:2.28 were finally selected to synthesize MoS_2_/GO NSs. Moreover, since TMB can be oxidized to a blue product in the exist of catalyst, POD-like activities of GO, MoS_2_, and MoS_2_/GO NSs were detected by TMB-H_2_O_2_ reaction (Additional file 1: Fig. S3) [18–20]. The results manifested that MoS_2_/GO NSs possessed higher POD-like activities than GO and MoS_2_ NSs, which were consistent with previous evidence about MoS_2_/GO [21]. Reportedly, MoS_2_ NSs-based system exhibited intrinsic antioxidant properties including POD-, superoxide dismutase (SOD)-, and catalase (CAT)-like activities, which could efficiently scavenge ROS including superoxide anion (O_2_^·−^), hydroxyl radicals (·OH), and H_2_O_2_ [18, 22]. Here, we proved that MoS_2_/GO NSs exhibited the highest catalytic efficiency. The detailed mechanism should be that, GO NSs have high vacancy defects and hole defects because of their over oxidation [23], the defects enhance the rapid electron transfer as well as high conductivity after reacting with MoS_2_, resulting in the high catalytic ability of MoS_2_/GO NSs [21, 22].

Furthermore, compared with PVA hydrogels at different weights in volume from 2.5% to 15% (w/v), compound hydrogels containing 10% (w/v) PVA exhibited the closest strength to the spinal cord and good adhesion (Additional file 1: Fig. S4). Therefore, 10% PVA was selected as the host hydrogels in the subsequent experiments. After being combined with GO, the zeta potential of MoS_2_/PVA changed from −10.5 mV to −33 mV (Additional file 1: Fig. S5), suggesting the enhancement of MoS_2_/GO/PVA stability.

Hydrogels could promote the proliferation of neural stem cells at the elastic modulus of 183-19700 Pa, and the lower the elastic modulus, the higher the proliferation rate [24]. As shown in Fig. 1i, the elastic modulus of experimental groups were 2361.99 Pa for PVA, 1500.24 Pa for GO/PVA, 1923.69 Pa for MoS_2_/PVA, and 220.07 Pa for MoS_2_/GO/PVA, which were all in the range of 183-19700 Pa. Notably, the elastic modulus of MoS_2_/GO/PVA is significantly lower (p < 0.05) than that of PVA, GO/PVA and MoS_2_/PVA. Previous studies showed that the mechanical properties of PVA could be enhanced by adding conductive nanoparticles [25], the lower elastic modulus may reduce the growth resistance of the spinal cord after SCI [24].

Electrical signaling among nerve cells is important for the maintenance of the nervous system and the recovery of the musculoskeletal motor system [26]. As shown in Fig. 1j, the average electrical conductivity of PVA, GO/PVA, MoS_2_/PVA, and MoS_2_/GO/PVA were 0.025, 0.028, 0.131, and 0.220 S/m, the average electrical conductivity of MoS_2_/PVA and MoS_2_/GO/PVA were significantly higher than that of GO/PVA and PVA (p < 0.05). Previous literature reported that the electrical conductivity of spinal cord tissue is between 0.08 and 0.23 S/m [27], the synthesized MoS_2_/GO/PVA with good conductivity is favorable for the connection of neurons, which is beneficial for the further repair of SCI. Moreover, we have detected the photoelectric effect of MoS_2_/GO and GO (Additional file 1: Fig. S7). In the cycle of 40 s light stimulation, the instantaneous current of MoS_2_/GO NSs could reach 5.2 × 10^–5^ A, while GO NSs were not detected significant current. The result indicated that MoS_2_/GO NSs play a key role in the electrical conductivity of the MoS_2_/GO/PVA, this could be related to the well semiconductor properties of MoS_2_ [28]. Additionally, improved conductivity of MoS_2_/GO/PVA might be attributed to increased two-dimensional order of MoS_2_/GO NSs caused by the combination of MoS_2_ and GO NSs [29].

The swelling rates of GO/PVA, MoS_2_/PVA, MoS_2_/GO/PVA, and PVA nanocomposite hydrogels were 2.55 for PVA, 2.51 for GO/PVA, 2.80 for MoS_2_/PVA, and 2.42 for MoS_2_/GO/PVA after 24 h, respectively (Fig. 1k). There was no significant difference in swelling rate among the four hydrogels, which indicated that their capacities of transporting small molecules were similar [30].

The degradation of the nanohydrogels was studied in the SBF for about six weeks. The PVA, MoS_2_/PVA, GO/PVA, and MoS_2_/GO/PVA hydrogels exhibited a similar trend in SBF solutions. As shown in Additional file 1: Fig. S8, they degraded rapidly in the first week (up to 40%), then the degradation trend to slow and all the hydrogels tended to degrade about 70% in the 42 days.

### Biosafety of nanocomposite hydrogels in vitro

**Fig. 2:**
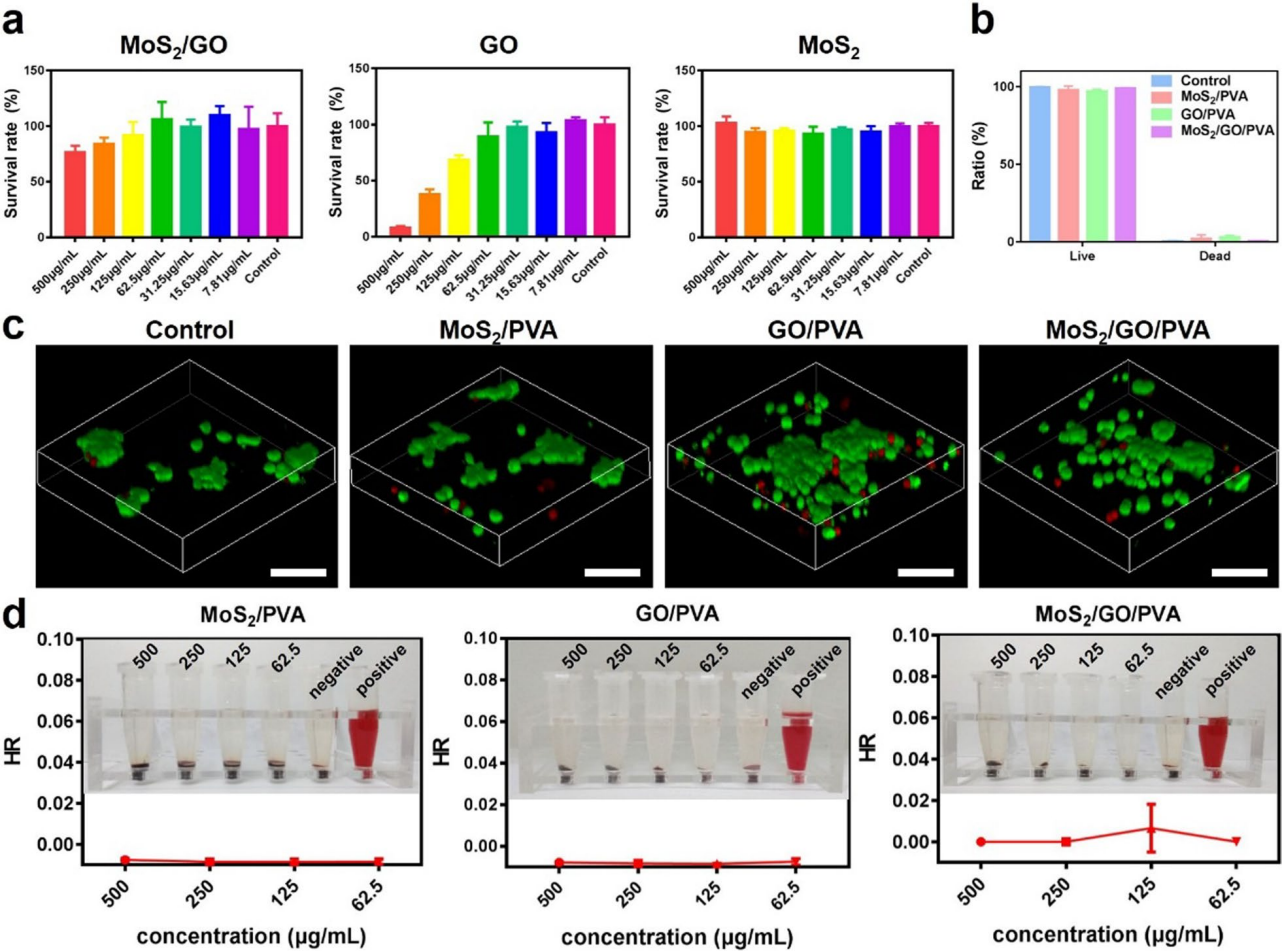
Cytocompatibility and blood compatibility of different hydrogels. **a** Cytotoxicity of NE-4C co-cultured with hydrogels with different concentrations of MoS_2_, GO, and MoS_2_/GO NSs at 24 h. **b** The ratio of live and dead cells in each group. **c** Live/Dead staining of NE-4C cells in different groups (Control, GO/PVA, MoS_2_/PVA, and MoS_2_/GO/PVA hydrogels). Live NE-4C cells were stained with calcein (green), the dead cells were stained with propidium iodide (red). Scale bar = 100 μm. **d** The hemolysis rate of MoS_2_/PVA, GO/PVA, and MoS_2_/GO/PVA hydrogels

Good biocompatibility is the most basic condition for the application of biomaterials [31]. We assessed the cytotoxicity of GO/PVA, MoS_2_/PVA, and MoS_2_/GO/PVA toward NE-4C by using CCK-8 (Fig. 2a). When their concentrations were below 62.5 µg/mL, the survival rates of GO/PVA, MoS_2_/PVA, and MoS_2_/GO/PVA groups were all above 85%, exhibiting low cytotoxicity. Thus, MoS_2_/GO, GO, and MoS_2_ at concentrations of 62.5 µg/mL were selected for the subsequent experiments. The NE-4C cell proliferation was further detected using CCK-8 assay in hydrogels of different components, and there was no significant difference in the proliferation rate of different hydrogels after 24 h of coculture (Additional file 1: Fig. S9). Live/dead staining was further applied in NE-4C to verify the cytotoxicity of GO/PVA, MoS_2_/PVA, and MoS_2_/GO/PVA (Fig. 2b, c). After cocultured with the above materials for 24 h, almost all the NE-4C cells were alive (green fluorescence) on the hydrogels, and the quantitative analysis of living and dead cells further indicated their good biocompatibility.

Blood compatibility is important to evaluate the biocompatibility of biomaterials [32]. As shown in Fig. 2d, the supernatant of all material groups and negative control (phosphate buffer saline, PBS) groups were clear, and they did not show obvious red color. In contrast, the supernatant of the positive control (double distilled water, dd H_2_O) turned distinct red color due to the release of hemoglobin from the broken red blood cells. The hemolysis rates of each material group were all significantly lower than 5%.

### Effects of nanocomposite hydrogels on neural cells differentiation

**Fig. 3:**
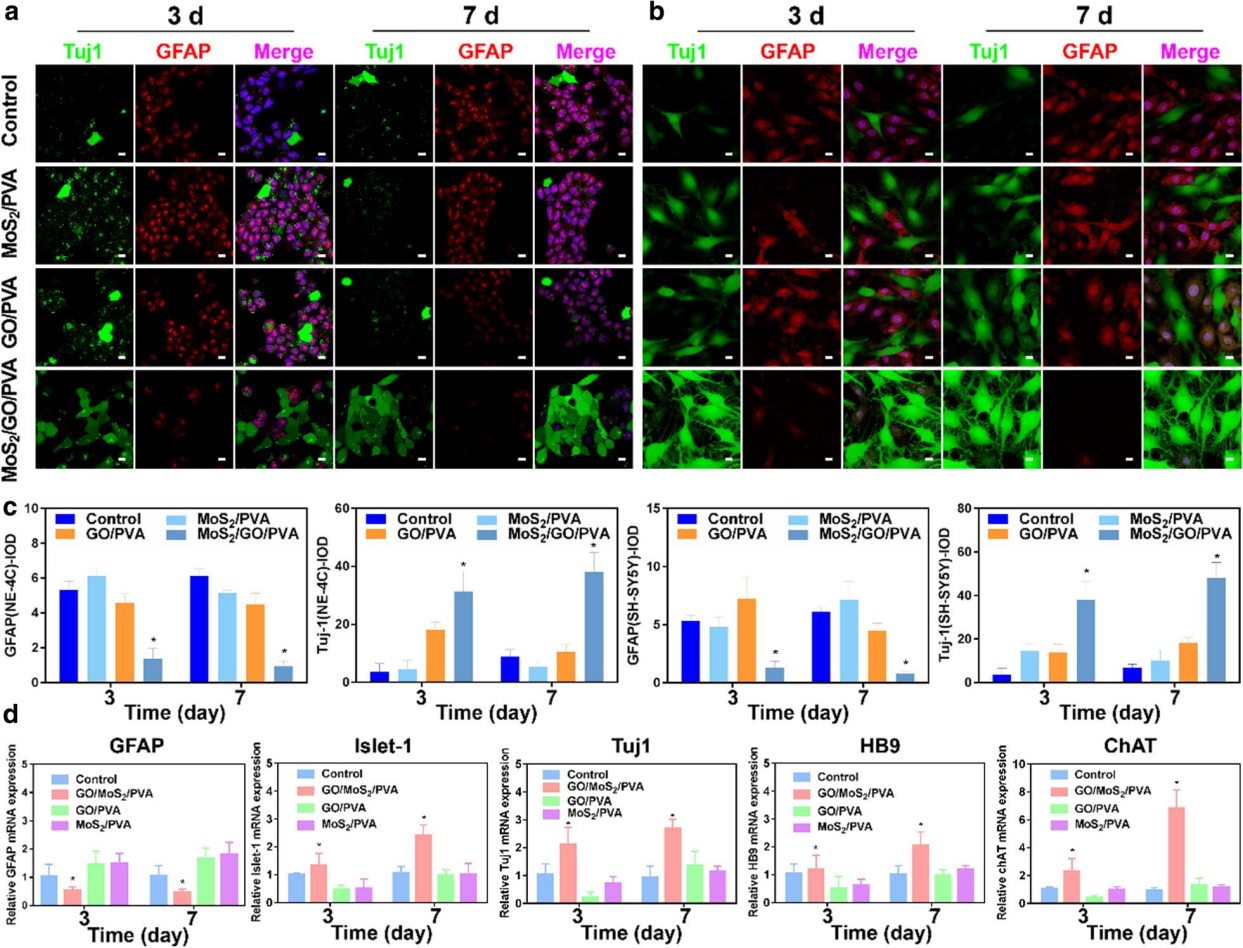
The growth and differentiation of NE-4C and SH-SY5Y cells after cocultured with PVA, GO/PVA, MoS_2_/PVA, and MoS_2_/GO/PVA hydrogels in vitro. Confocal microscopic images of (**a**) NE-4C cells and **b** SH-SY5Y cells. Tuj1-positive cells (green) and GFAP-positive cells (red) in different groups (Control, GO/PVA, MoS_2_/PVA, and MoS_2_/GO/PVA hydrogels) at 3 and 7 days. All nuclei were stained with DAPI (blue). Scale bar = 50 μm. **c** Fluorescence intensity values corresponding to confocal photos of each group. **d** Quantitative of relative gene expression levels of Tuj1, GFAP, ChAT, Islet-1, and HB9 in NE-4C cells in different groups (Control, GO/PVA, MoS_2_/PVA, and MoS_2_/GO/PVA hydrogels) at 3 and 7 days. Error bars indicate the SD from four different experiments; *p < 0.05, **p < 0.01, all groups were compared with the control group

Encouraged by the excellent biosafety of the composite hydrogels, we further studied their effects on neural cells growth and differentiation, which is highly related to the process of spinal cord repair [33]. Neural stem cells [34] and neuroblastoma cells [35] both have the potential to differentiate into both nerve cells and glial cells. In order to verify the differentiation effect of nanohydrogels on neural cells of different species. NE-4C and SH-SY5Y cells were cocultured with nanohydrogels to detect their direction of differentiation. Here, neuronal-specific marker protein class III beta tubulin (Tuj1) and astrocytes specificity of collagen fiber acidic protein (GFAP) were applied to detect the fate of neural cells differentiation after cocultured with different hydrogels. By confocal microscope (Fig. 3a–c), we found that the expression of neuron-specific Tuj1 in control, GO/PVA, and MoS_2_/PVA groups were significantly lower than MoS_2_/GO/PVA group. In addition, we also found that Tuj-1 positive SH-SY5Y cells in the MoS_2_/GO/PVA group grew a large number of tentacles in all directions, while this phenomenon was not found in other groups. Glial cells are the main components of nerve scars and important factors that hinder the repair of nerve tissue [36]. GFAP is the main marker of glial cells, and we next investigated the expression of GFAP among all groups. As expected, MoS_2_/GO/PVA inhibited the expression of GFAP, and the red fluorescence in the MoS_2_/GO/PVA group was weaker than other groups after 3 days and 7 days. It was found that MoS_2_/GO/PVA could promote the differentiation of neural stem cells into neural cells more effectively than other hydrogels. Interestingly, the conductivity of MoS_2_/GO/PVA was also the highest among all groups. Previous studies have also shown the close relationship between neural differentiation and electrical conductivity of the cells [37, 38]. Moreover, their excellent biocompatibility matched Young’s modulus, and ROS quenching ability may also benefit neural differentiation. Therefore, this test indicated that MoS_2_/GO/PVA composite hydrogels have great advantages in promoting nerve cells growth and differentiation.

We further examined the represent genes changes of NE-4C cells in different treatment groups. As shown in Fig. 3d, Tuj1, Choline acetyltransferase (ChAT), motor neuron markers Islet-1, and the HB9 expression in MoS_2_/GO/PVA hydrogel group were significantly higher (p < 0.05) than that on other groups. In contrast, the expression of GFAP in the MoS_2_/GO/PVA hydrogel group was significantly decreased (p < 0.05) than in other groups. Overall, the quantitative reverse transcription polymerase chain reaction (RTqPCR) test on Tuj1, ChAT, Islet-1, and the HB9 expression further confirmed the promotion of nerve growth factor and inhibition of the glia growth factor, which was in accord with the fluorescence images. The above results indicated that MoS_2_/GO/PVA hydrogel promoted the NE-4C cells to differentiate into nerve cells and inhibited glial cell differentiation, which may attribute to MoS_2_/GO/PVA hydrogel’s characteristics of high conductivity [39] and low elastic modulus [24].

### Inhibitory effects of nanocomposite hydrogels on the secondary injury of SCI

**Fig. 4:**
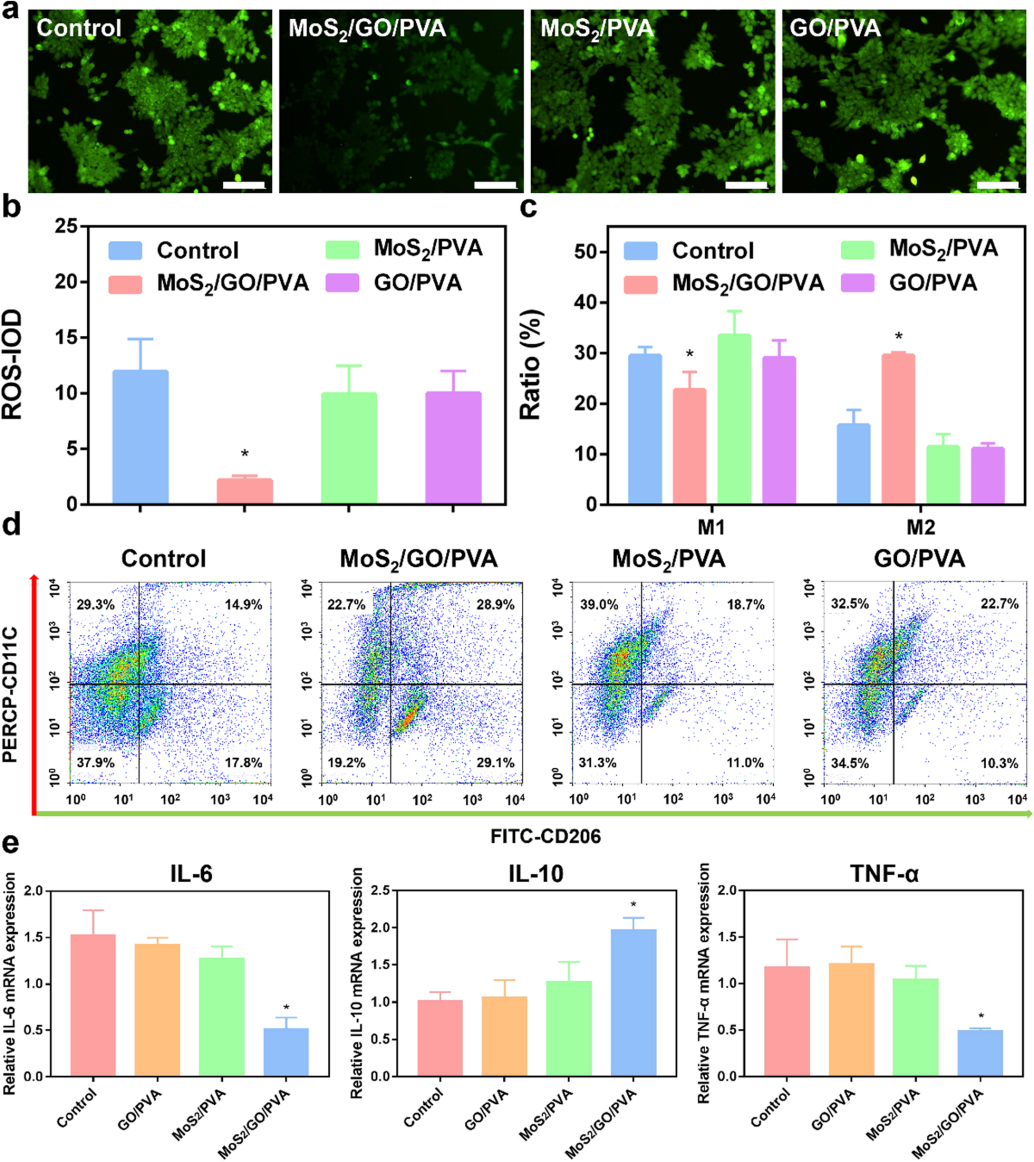
ROS scavenging and anti-inflammatory properties of MoS_2_/GO/PVA hydrogel. **a** The effect of PVA (set as control), GO/PVA, MoS_2_/PVA, and MoS_2_/GO/PVA hydrogels on ROS production in H_2_O_2_-induced NE-4C cells. Scale bar = 50 μm. **b** DCF fluorescence intensity of PVA (set as control), GO/PVA, MoS_2_/PVA, and MoS_2_/GO/PVA hydrogels. **c** The M1, M2 macrophage ratio in each group. **d** The flow cytometry images of RAW264.7 cells after cultured with PVA (set as control), GO/PVA, MoS_2_/PVA, and MoS_2_/GO/PVA hydrogels for 24 h. **e** RT-PCR results of IL-6, IL-10, and TNF-α in different treatment groups; *p < 0.05. **p < 0.01. All groups were compared with the control group

ROS is one of the major factors produced in the secondary injury after SCI. In order to test the effects of composite hydrogel on the secondary injury after SCI, we tested the ROS level using dichloro-dihydro-fluorescein diacetate (DCFH-DA) in NE-4C cells. In Fig. 4a and b, the NE-4C cells in the MoS_2_/GO/PVA group showed the weakest fluorescent intensity and lowest ROS level compared with other groups, we used the same method to detect the effect of hydrogels on ROS levels in RAW264.7 cells, and the results were consistent with those in NE-4C cells (Additional file 1: Fig. S10), indicating that the MoS_2_/GO/PVA hydrogel could effectively inhibit the production of ROS.

Macrophages begin to increase rapidly in the SCI part after 1–2 days due to the serious inflammatory [40]. Their polarization ratio is highly related to SCI recovery. Here, we employed flow cytometry to detect the proportion of macrophages with different polarization directions after cocultured with different hydrogel groups (Fig. 4c and d). Macrophages were in a state of inflammation after being stimulated by LPS, then after cocultured with nanohydrogel for 24 h, MoS_2_/GO/PVA group showed the highest M2 ratio in Fig. 4c, indicating that MoS_2_/GO/PVA nanohydrogel can effectively reverse the transformation of M1 into M2, thus inhibiting the secondary injury caused by the excessive concentration of M1 in SCI.

We next detected the inflammatory cytokines, including IL-6, IL-10, and TNF-α, in different groups (Fig. 4e). The expression of protective cytokine IL-10 was relatively higher in the MoS_2_/GO/PVA group than the other three groups. In contrast, the pro-inflammatory mediators IL-6 and TNF-α decreased in the MoS_2_/GO/PVA group, indicating that the MoS_2_/GO/PVA hydrogel could inhibit the destructive inflammatory response.

### Effects of nanocomposite hydrogels on spinal cord repair in vivo

**Fig. 5:**
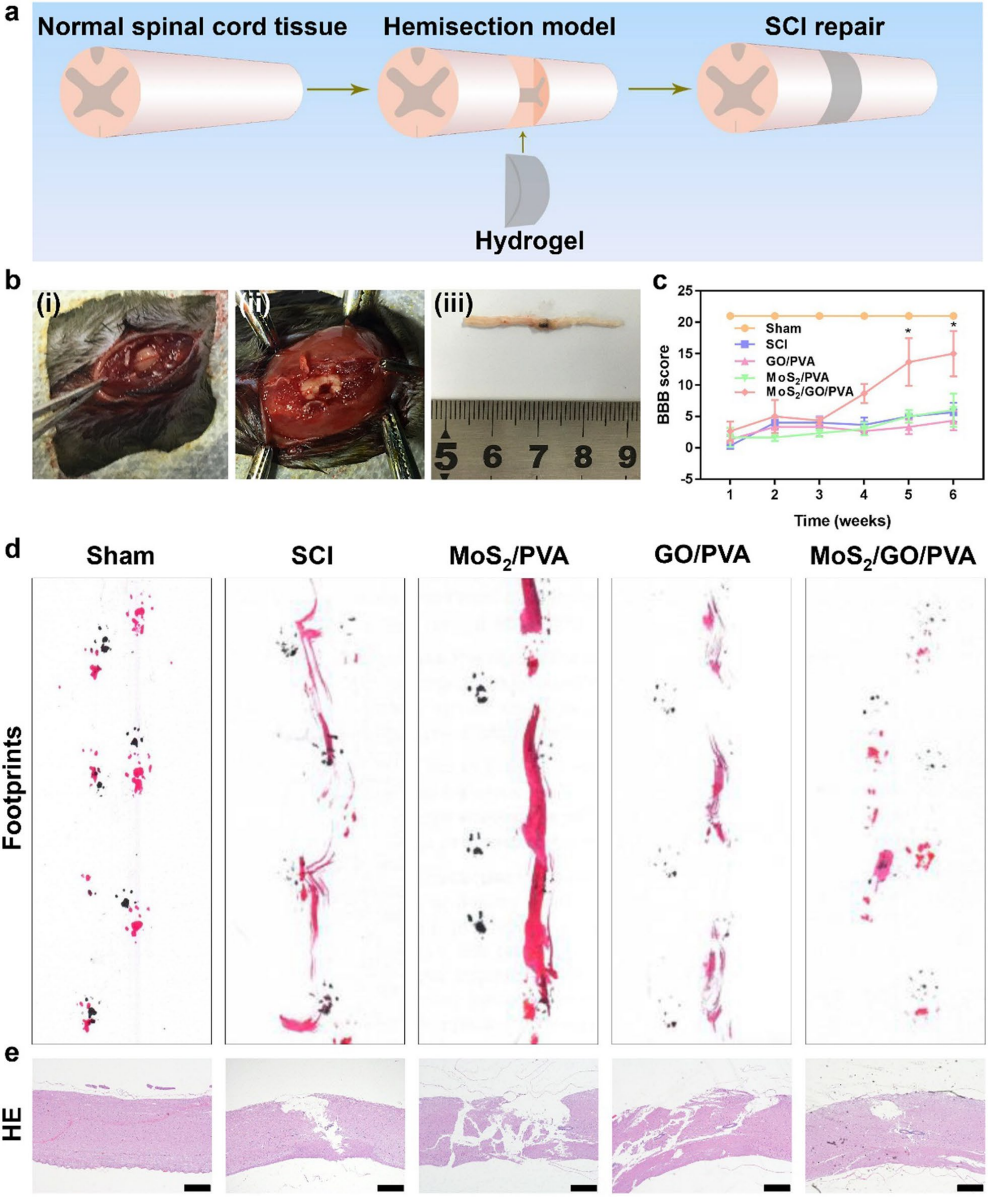
The treatment effects of different hydrogels on SCI mice. **a** Scheme of the surgical process of hemisected spinal cord repair with hydrogel. **b** Images of implanting hydrogels during SCI operation. (i) The spinal cord was exposed intra-operatively. (ii) A nanocomposite hydrogel was implanted in SCI intra-operatively. (iii) The spinal cord specimen was obtained at 6 weeks after surgery. **c** BBB scores of mice at 6 weeks after surgery. n = 6 animals in each group. Error bars represent SD. *p < 0.05; **p < 0.01. All groups were compared with the SCI group. **d** Representational footprints of Sham, SCI, GO/PVA, MoS_2_/PVA, and MoS_2_/GO/PVA groups. The mouse front paw footprints were black, and the hind paw footprints were red. **e** Representative H&E images of spinal cords of Sham, SCI, GO/PVA, MoS_2_/PVA, and MoS_2_/GO/PVA groups at 6 weeks postoperation, Scale bar = 50 μm

Restoring the electrical conductivity of the spinal cord quickly after SCI will benefit the functional recovery of the spinal cord innervated area [41]. Therefore, we filled the hemisection of the spinal cord in mice with composite hydrogels to study their prothetic effects on the SCI and the subsequent influence on the motor function dominated by the injured area of the spinal cord (Fig. 5a, b). Six weeks after the SCI and transplantation, inflammatory or toxic reactions were not observed in the major visceral organs in all groups. The H&E results indicated that hydrogels transplantation did not cause harm to the main organs of all groups (Additional file 1: Fig. S11), which was also consistent with the results of the in vitro safety studies.

The anterior cord of the spinothalamic descends ipsilaterally and innervates the motor function of the ipsilateral soma [42]. Therefore, ipsilateral motor dysfunction presented in spinal cord hemisection injury. The BBB scale is a classical scale to evaluate the recovery of motor function in laboratory mice [43]. Therefore, we monitored the recovery of motor function of mice after SCI by evaluating BBB scores once a week for 6 weeks after surgery (Fig. 5c). We observed that in the Sham group, motor function remained normal, with a score of 21 through 6 weeks. The SCI, GO/PVA, and MoS_2_/PVA groups had a slight increase after 6 weeks in postoperative BBB scores, but the scores were still below 10 points, and there was no statistical difference among the three groups (Additional file 2: Video S1). But, the scores in the MoS_2_/GO/PVA group at week 4 and week 5 were significantly higher than those of SCI, GO/PVA, and MoS_2_/PVA groups. The results illustrated that the MoS_2_/GO/PVA hydrogel could accelerate the recovery of hind limb motor function of mice after SCI. In addition to scores, footprints can also be observed to promote the motor function of mice after SCI (Fig. 5d). At the 6th week after surgery, drag traces and directional instability traces were found in SCI, GO/PVA, and MoS_2_/PVA groups. In contrast, the mice in the MoS_2_/ GO/PVA group showed clear footprints, which further demonstrated the promoting effects of MoS_2_/GO/PVA hydrogel on the recovery of hind limb motor function in mice. As shown in Fig. 5e 6 weeks after the transplantation, GO/PVA, MoS_2_/PVA, and MoS_2_/GO/PVA groups showed partial spinal cord repair compared to the SCI group. It is noteworthy that in the MoS_2_/GO/PVA group, the nanocomposite hydrogel adhered well to the spinal cord during specimen collection without apparent inflammatory reaction around the material.

**Fig. 6:**
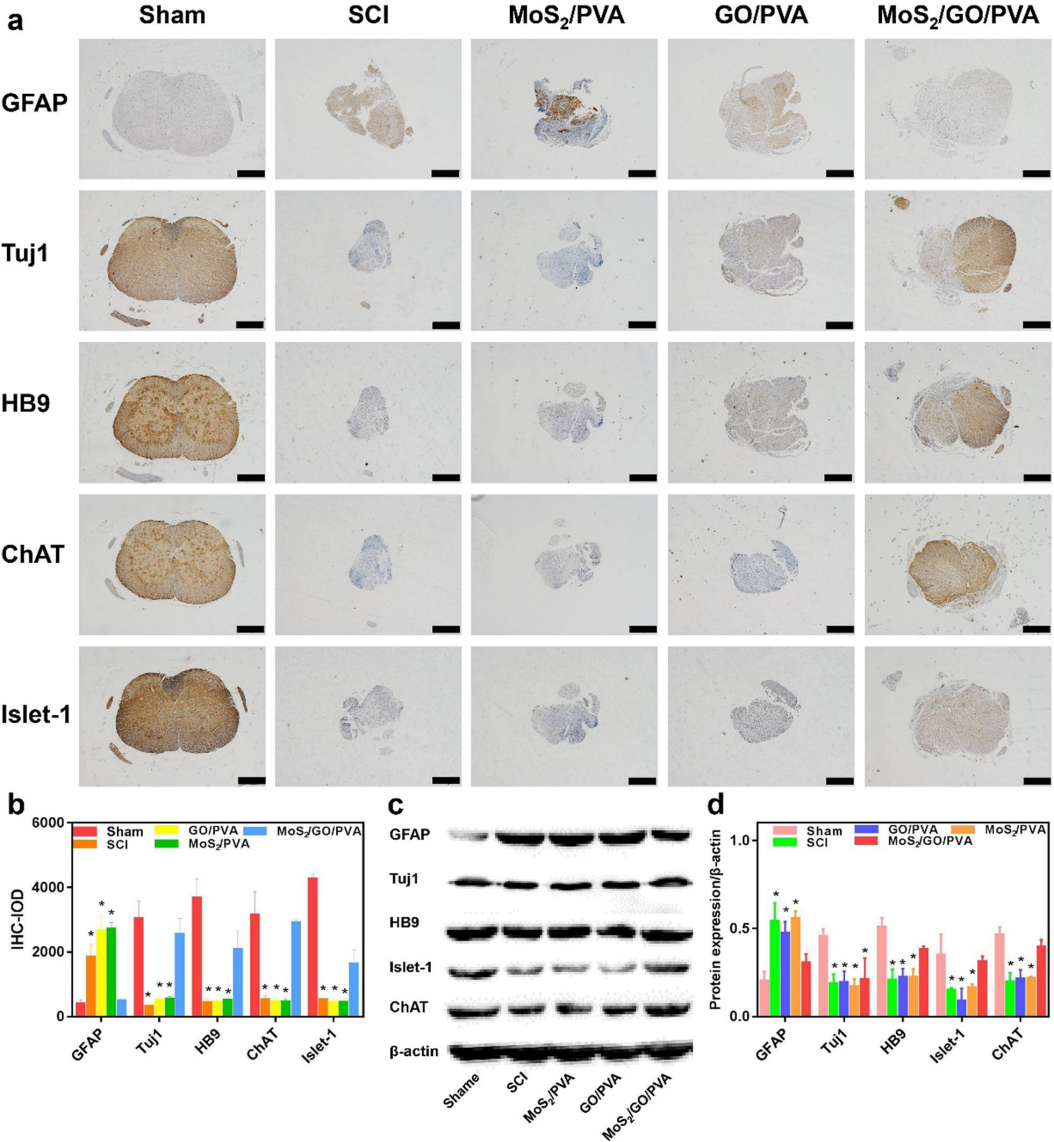
Differentiation direction of spinal cord tissue in each group. **a** Representational IHC images of Tuj1, GFAP, ChAT, Islet-1, and HB9 in Sham, SCI, GO/PVA, MoS_2_/PVA, and MoS_2_/GO/PVA groups. The spinal cord tissue of the original defect site was stained with IHC. Due to the difference in the degree of spinal cord repair in each group, there were differences in the area and morphology of immunohistochemistry sections in each group. Scale bars = 50 μm. **b** The IOD value of the IHC images in each group; *p < 0.05, **p < 0.01. All groups were compared with Sham group. **c** Western blotting bands of Tuj1, GFAP, ChAT, Islet-1, and HB9 of the NE-4C cells in Sham, SCI, GO/PVA, MoS_2_/PVA, and MoS_2_/GO/PVA groups at 6 weeks postoperatively. **d** Gray values of western blotting in each group; *p < 0.05. All groups were compared with the Sham group

In order to further verify the differentiation direction of spinal cord tissue repair, we tested the spinal cord tissues using immunohistochemistry (IHC) (Fig. 6a, b) and western blotting (WB) (Fig. 6c, d). The expression of glial-related protein GFAP was relatively lower in the sham and MoS_2_/GO/PVA group, while the expression of neurons-related protein Tuj1, HB9, Islet-1, and ChAT was significantly higher than the other groups. In addition, the glia marker GFAP was mainly expressed in the SCI group, GO/PVA group, and MoS_2_/PVA group. The results were consistent with the experimental results of neural stem cell differentiation in vitro*,* indicating that MoS_2_/GO/PVA could promote neural tissue differentiation in vivo*.*

## Conclusion

In summary, we have successfully developed a sort of highly conductive, soft, excellent biocompatible, and anti-inflammatory nanocomposite hydrogel. SCI could be effectively repaired by the composite hydrogel because of its appropriate mechanical property and excellent electrical conductivity, which could quickly restore the continuity and electrical conductivity after SCI. More importantly, the composite hydrogel could promote NE-4C to neuronal differentiation and inhibit the development of astrocytes. The hydrogel exhibit has an excellent anti-oxidative property, which could effectively attenuate the ROS and reduce the M1/M2 macrophage ratio in vitro. Most importantly, the in vivo study also showed that the implantation of composite hydrogel could promote the repair of spinal cord tissue and improve the locomotor function of SCI mice. Hence, our works are of great importance for future biomaterials design and medicinal applications in SCI restoration.

## Supplementary Information

**Additional file 1: Fig. S1.** Mapping images of MoS_2_/GO CN. **Fig. S2.** XPS spectra of MoS_2_/GO CN. **Fig. S3.** POD-like properties of GO, MoS_2,_ and MoS_2_/GO NSs. **Fig. S4**. Adhesion tests of hydrogels to spinal cord tissue in vitro. **Fig. S5.** Zeta potential of nanocomposite hydrogels. **Fig. S6.** Conductivity test of nanocomposite hydrogels in vitro. **Fig. S7.** Photocurrent of PVA, GO/PVA, MoS_2_/PVA and MoS_2_/GO/PVA hydrogels. **Fig. S8.** The degradation of the nanocomposite hydrogels in vitro. **Fig. S9.** The proliferation rate of the nanocomposite hydrogels in vitro. **Fig. S10.** Effects of hydrogels on ROS level in RAW264.7 cells. **Fig. S11.** Safety test of nanocomposite hydrogels in vivo.

**Additional file 2: Video S1.** The motor function of each experimental group 6 weeks after the operation.

## Data Availability

All the original data are available upon reasonable request for correspondence authors.
