# Supplementary material for: Conducting molybdenum sulfide/graphene oxide/polyvinyl alcohol nanocomposite hydrogel for repairing spinal cord injury
Source: J Nanobiotechnology. 2022 May 6;20:210. doi: 10.1186/s12951-022-01396-8 (PMC9074236; doi:10.1186/s12951-022-01396-8)
Supplement: Supplementary file 1 — Additional file 1: Fig. S1. Mapping images of MoS2/GO CN. Fig. S2. XPS spectra of MoS2/GO CN. Fig. S3. POD-like properties of GO, MoS2, and MoS2/GO NSs. Fig. S4. Adhesion tests of hydrogels to spinal cord tissue in vitro. Fig. S5. Zeta potential of nanocomposite hydrogels. Fig. S6. Conductivity test of nanocomposite hydrogels in vitro. Fig. S7. Photocurrent of PVA, GO/PVA, MoS2/PVA and MoS2/GO/PVA hydrogels. Fig. S8. The degradation of the nanocomposite hydrogels in vitro. Fig. S9. The proliferation rate of the nanocomposite hydrogels in vitro. Fig. S10. Effects of hydrogels on ROS level in RAW264.7 cells. Fig. S11. Safety test of nanocomposite hydrogels in vivo. [file 12951_2022_1396_MOESM1_ESM.docx]

Additional file 1

**Conducting Molybdenum Sulfide/Graphene Oxide/ Polyvinyl Alcohol Nanocomposite Hydrogel for Repairing Spinal Cord Injury**

Lingling Chen ^1, †^, Wanshun Wang ^1,2, †^, Zefeng Lin ^1^, Yao Lu ^3,4^, Hu Chen ^1,3^, Binglin Li ^1^, Zhan Li ^1^, Hong Xia ^1,3,^*, Lihua Li ^5,^*, Tao Zhang ^1,3,^*

^1^ Guangdong Key Lab of Orthopedic Technology and Implant Materials, Key Laboratory of Trauma & Tissue Repair of Tropical Area of PLA, Orthopaedic Center, General Hospital of Southern Theater Command of PLA, Guangzhou 510010, Guangdong, China.

^2^ The Second Clinical Medical College, Guangzhou University of Chinese Medicine, Guangzhou 510405, Guangdong, China.

^3^ Southern Medical University, 1023 South Shatai Road, Guangzhou 510515, Guangdong, China.

^4^ Department of Orthopedics, Clinical Research Centre, Zhujiang Hospital, Southern Medical University, 253 Gongye Road, Guangzhou 510282, Guangdong, China.

^5^ Department of Applied Physics, The Hong Kong Polytechnic University, Kowloon 999077, Hong Kong, China.

*Correspondence: [gzxiahong2@126.com](mailto:gzxiahong2@126.com); [lihua361@126.com](mailto:lihua361@126.com); [gzlupus@126.com](mailto:gzlupus@126.com)

^†^ Lingling Chen and Wanshun Wang contributed equally to this work.

A full list of author information is available at the end of the article.

This Supplementary Information includes:

Fig. S1 Mapping images of MoS_2_/GO CN

Fig. S2 XPS spectra of MoS_2_/GO CN.

Fig. S3 POD-like properties of GO, MoS_2,_ and MoS_2_/GO NSs.

Fig. S4 Adhesion tests of hydrogels to spinal cord tissue in vitro.

Fig. S5 Zeta potential of nanocomposite hydrogels.

Fig. S6 Conductivity test of nanocomposite hydrogels in vitro.

Fig. S7 Photocurrent of PVA, GO/PVA, MoS_2_/PVA and MoS_2_/GO/PVA hydrogels.

Fig. S8 The degradation of the nanocomposite hydrogels in vitro.

Fig. S9 The proliferation rate of the nanocomposite hydrogels in vitro.

Fig. S10 Effects of hydrogels on ROS level in RAW264.7 cells.

Fig. S11 Safety test of nanocomposite hydrogels in vivo.

Video S1. The motor function of each experimental group 6 weeks after the operation.


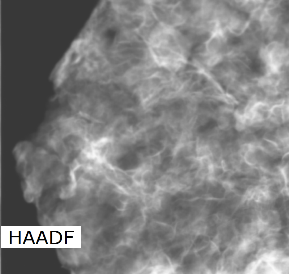

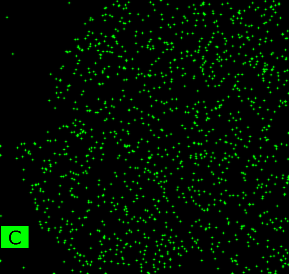

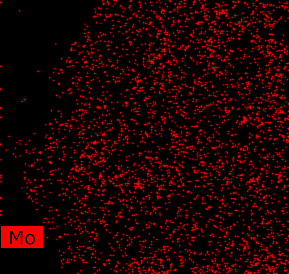

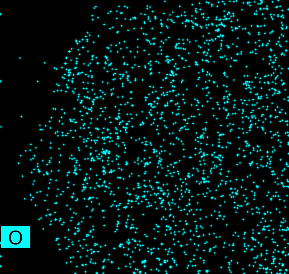

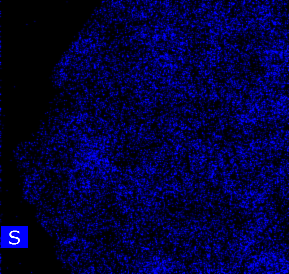

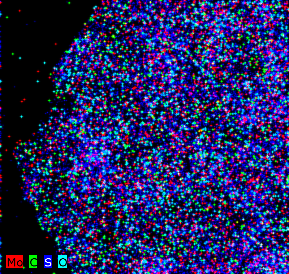


**Figure S1 Mapping images of MoS_2_/GO CN.** Mo, S, C, O mapping of the MoS_2_/GO CN displayed the elemental distribution of four elements in the CN, Scale bar = 400 nm.


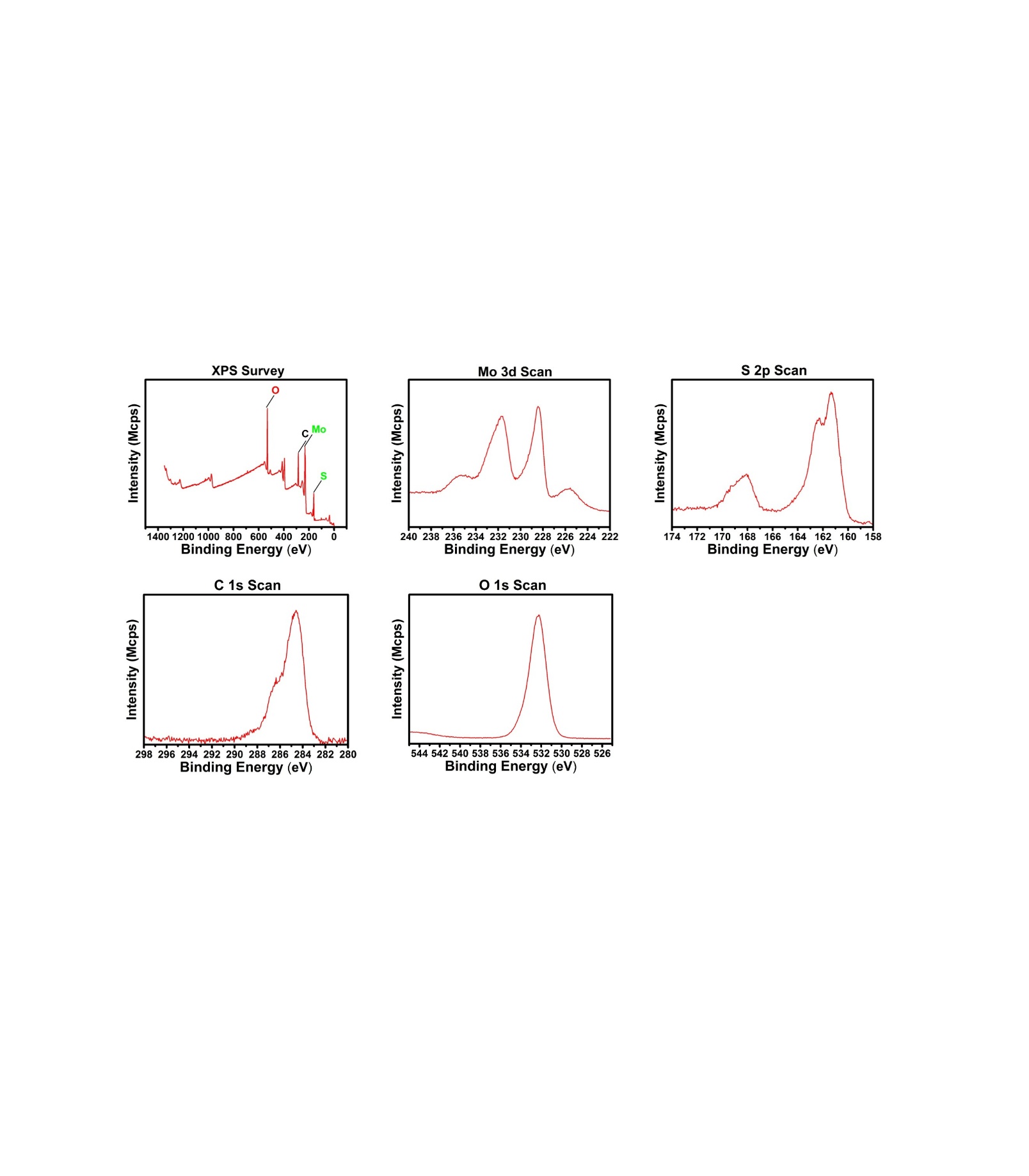


**Figure S2 XPS spectra of MoS_2_/GO CN.** The XPS spectrum of CN was further confirmed that Mo, S, C, and O in MoS_2_/GO CN. The XPS spectrum of Mo 3d, S 2p, C 1s, and O 1s displayed element valence states in MoS_2_/GO CN.

**
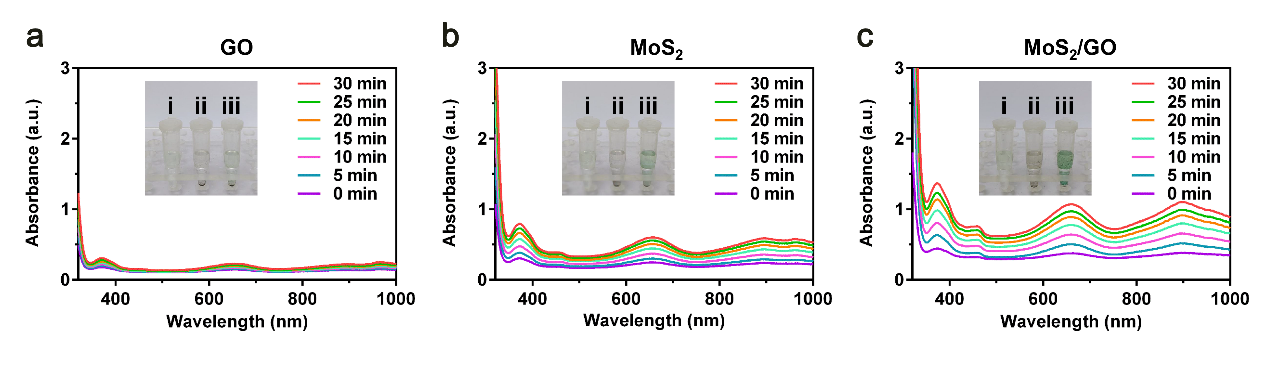
**

**Fig. S3 POD-like properties of GO, MoS_2,_ and MoS_2_/GO NSs.** POD-like activities of (a) GO, (b) MoS_2,_ and (c) MoS_2_/GO NSs at different time intervals (0, 5, 10, 15, 20, 25, 30 min). Inset: (i) TMB + H_2_O_2_, (ii) TMB + NSs, (iii) TMB + H_2_O_2_ + GO or MoS_2_ or MoS_2_/GO NSs. The MoS_2_/GO NSs + H_2_O_2_ group exhibits typical oxidation peaks of TMB with increasing time, and the solution becomes endowed with a blue color, indicating the POD-like activity of MoS_2_/GO NSs.


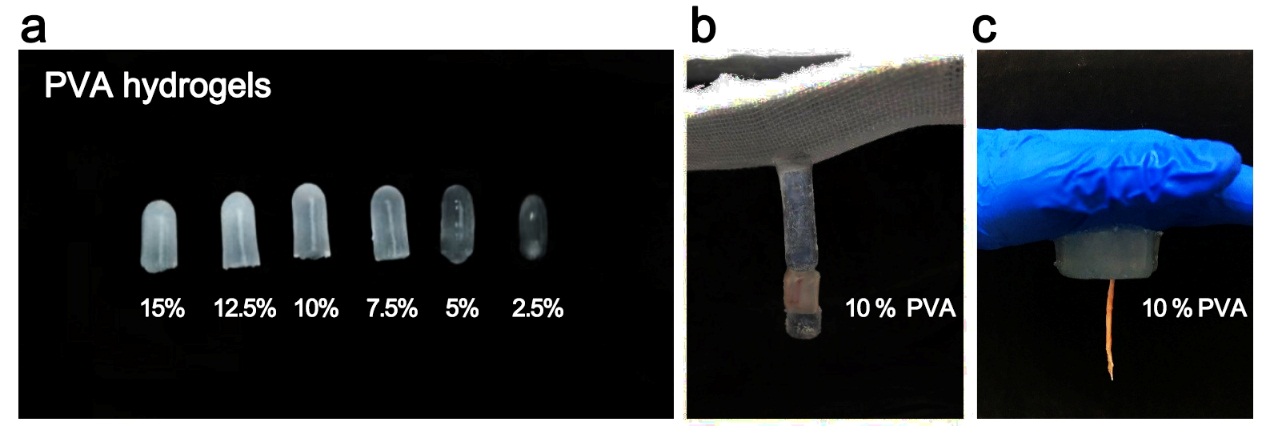


**Figure S4 Adhesion tests of hydrogels to spinal cord tissue in vitro.** (a) The general image of PVA hydrogels at different weights in volume from 2.5% to 15% (w/v). (b, c) 10% (w/v) PVA hydrogels could adhere firmly to spinal cord tissue, gauze, and gloves and could withstand the action of gravity.


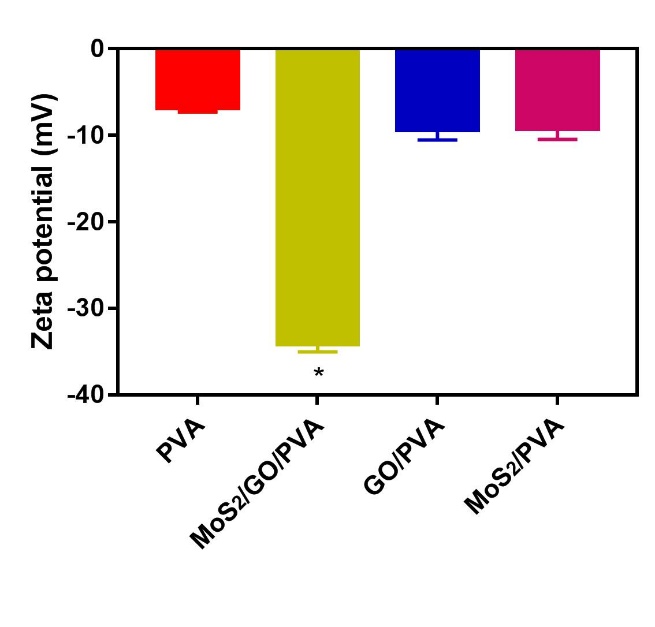


**Figure S5 Zeta potentials of PVA, MoS_2_/GO/PVA, GO/PVA, and MoS_2_/PVA.** The Zeta negative potential of MoS_2_/GO/PVA was significantly higher than that of the other three groups.


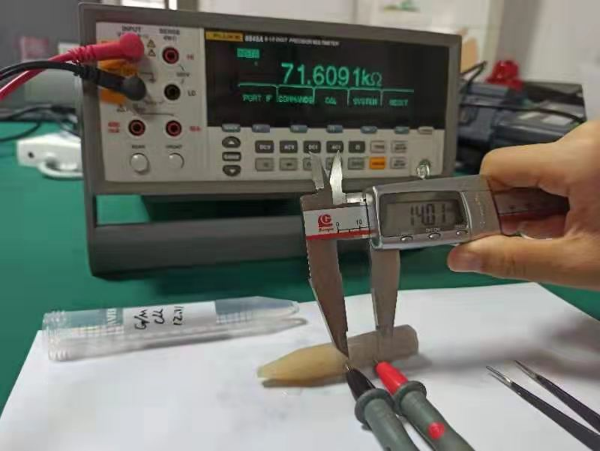


**Figure S6 Conductivity test of nanocomposite hydrogels in vitro.** Digital multimeter detected the conductivity of hydrogels and calculated the conductivity of hydrogels.


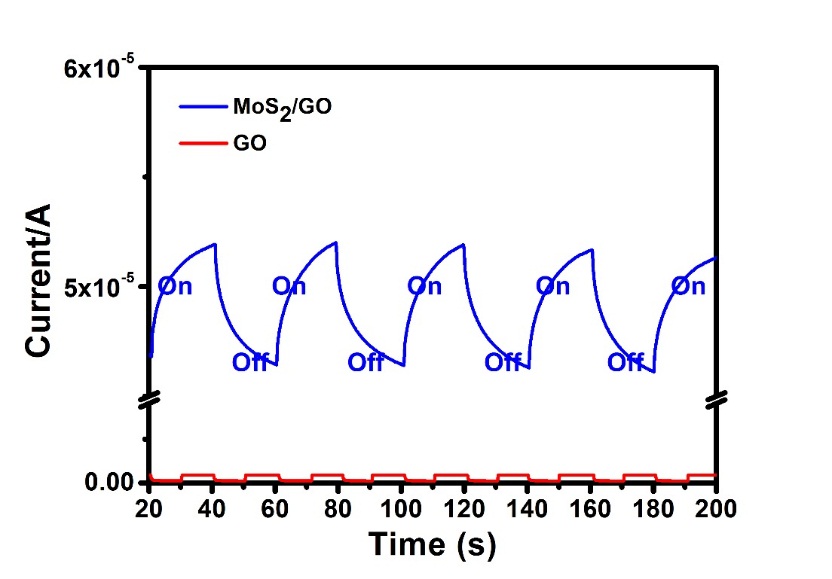


**Figure S7 Photocurrent of GO, MoS_2_/GO nanoparticles.** MoS_2_/GO nanomaterial can be observed significant current.


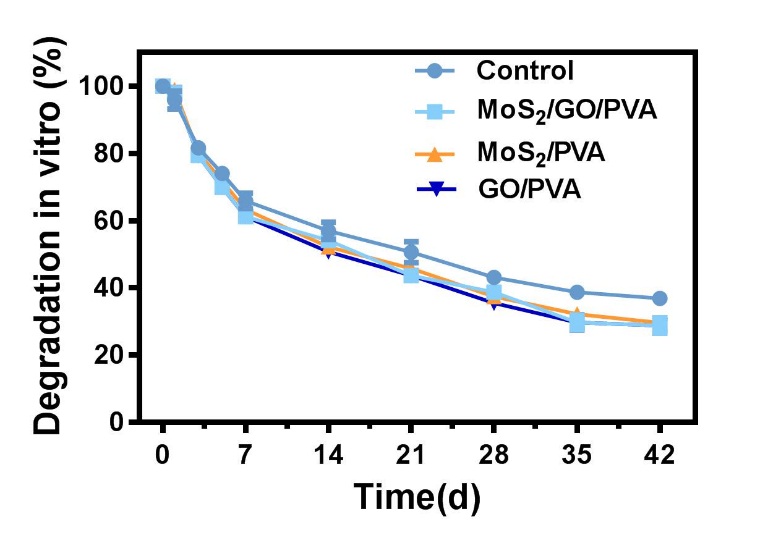


**Figure S8 The degradation of the nanocomposite hydrogels in vitro.** All nanocomposite hydrogels degraded about 70% in the simulated body fluid during six weeks.


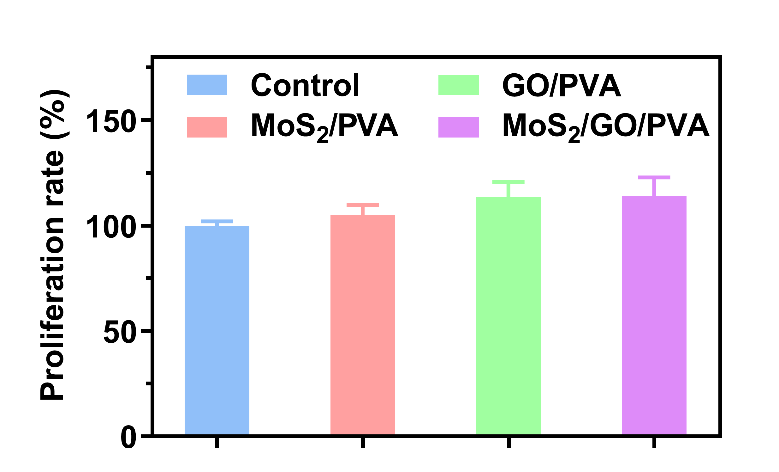


**Figure S9** **The proliferation rate of the nanocomposite hydrogels in vitro.** The NE-4C cells proliferation rate of different hydrogels had no significant difference after 24 h of coculture.


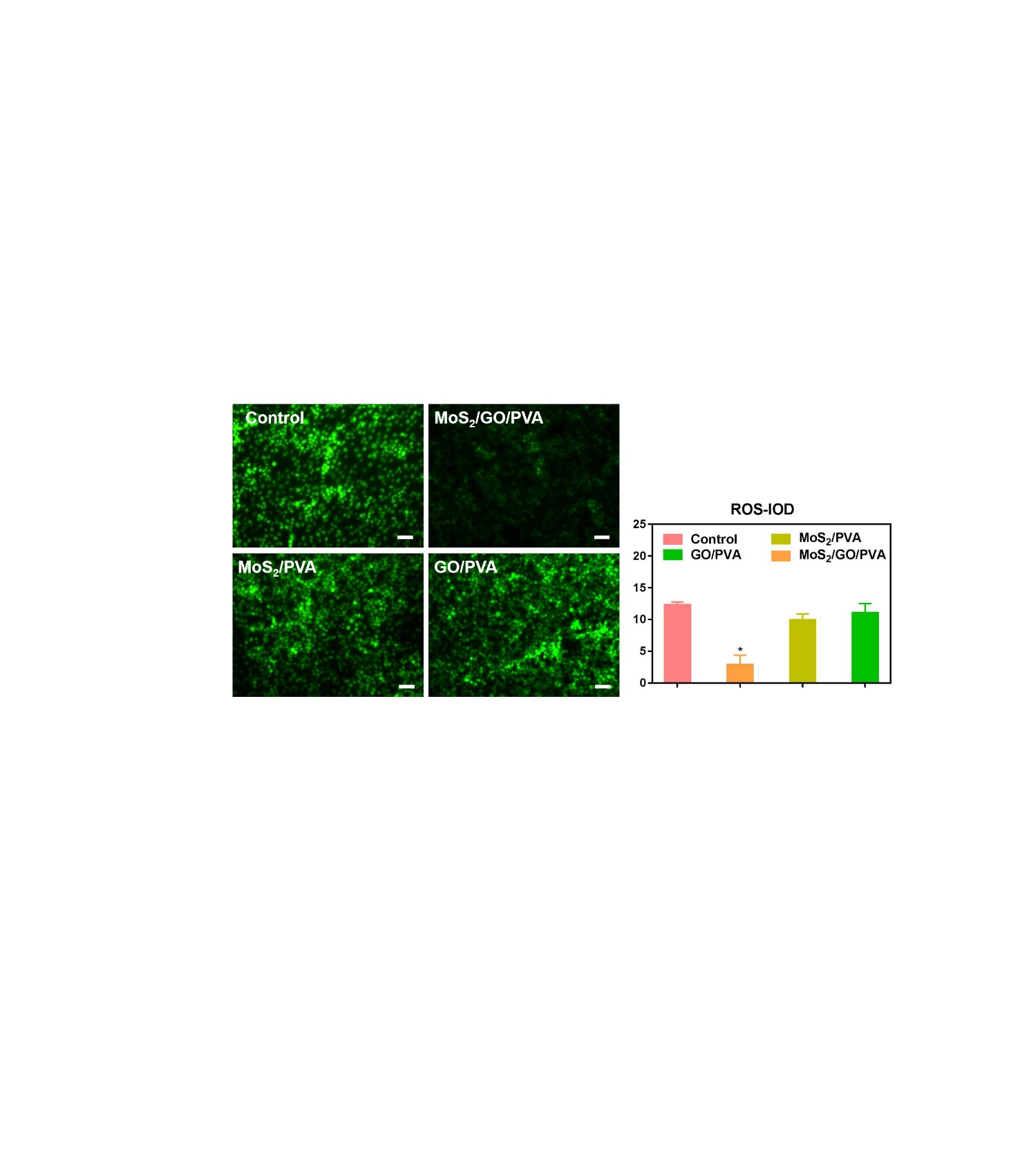


**Figure S10** **Effects of hydrogels on ROS level in RAW264.7 cells.** The ROS level in MoS_2_/GO/PVA group was significantly lower than that in other groups. Scale bar = 50 μm.

**
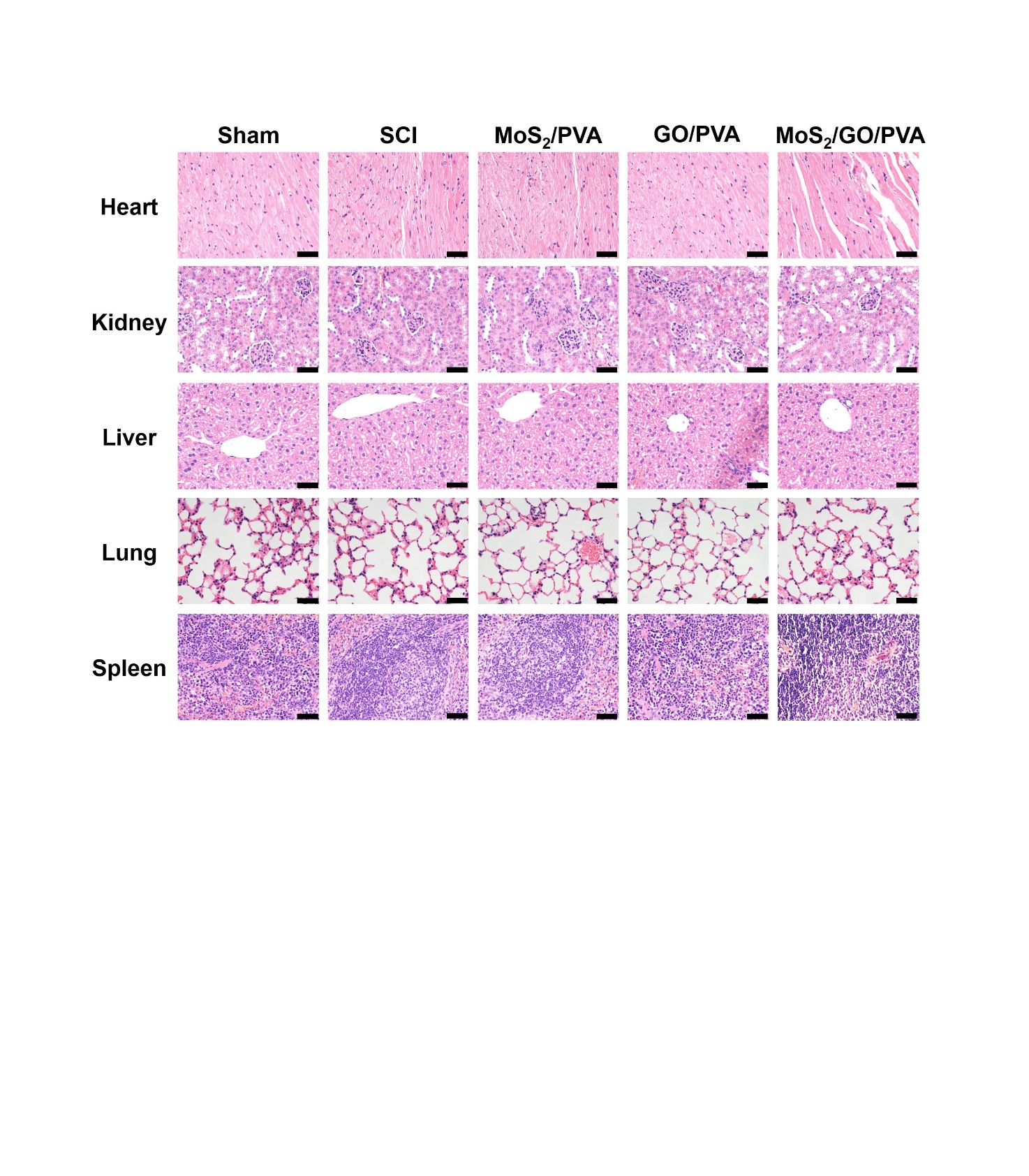
**

**Figure S11 Safety test of nanocomposite hydrogels in vivo.** There were no obvious toxic and side effects in the main visceral tissues of animals in all experimental groups. Scale bar = 50 μm.

**Video S1. The motor function of each experimental group 6 weeks after the operation.** The left hind limbs motor function of mice in the MoS_2_/GO/PVA group was significantly better than that in groups GO/PVA, MoS_2_/PVA, and SCI.
